# Role of Chemokines in the Development and Progression of Alzheimer’s Disease

**DOI:** 10.1007/s12031-022-02047-1

**Published:** 2022-07-12

**Authors:** Jakub Wojcieszak, Katarzyna Kuczyńska, Jolanta B. Zawilska

**Affiliations:** 1 Department of Pharmacodynamics, Medical University of Lodz, Muszynskiego 1, 90-151 Lodz, Poland; 2 Department of Neuroscience Care and Society, Division of Neurogeriatrics, Karolinska Institutet, 17164 Solna, Sweden

**Keywords:** Alzheimer’s disease, Amyloid β, Tau, Neuroinflammation, Chemokines, Chemokine receptors

## Abstract

Alzheimer’s disease (AD) is a progressive neurogenerative disorder manifested by gradual memory loss and cognitive decline due to profound damage of cholinergic neurons. The neuropathological hallmarks of AD are intracellular deposits of neurofibrillary tangles (NFTs) and extracellular aggregates of amyloid β (Aβ). Mounting evidence indicates that intensified neuroinflammatory processes play a pivotal role in the pathogenesis of AD. Chemokines serve as signaling molecules in immune cells but also in nerve cells. Under normal conditions, neuroinflammation plays a neuroprotective role against various harmful factors. However, overexpression of chemokines initiates disruption of the integrity of the blood–brain barrier, facilitating immune cells infiltration into the brain. Then activated adjacent glial cells–astrocytes and microglia, release massive amounts of chemokines. Prolonged inflammation loses its protective role and drives an increase in Aβ production and aggregation, impairment of its clearance, or enhancement of tau hyperphosphorylation, contributing to neuronal loss and exacerbation of AD. Moreover, chemokines can be further released in response to growing deposits of toxic forms of Aβ. On the other hand, chemokines seem to exert multidimensional effects on brain functioning, including regulation of neurogenesis and synaptic plasticity in regions responsible for memory and cognitive abilities. Therefore, underexpression or complete genetic ablation of some chemokines can worsen the course of AD. This review covers the current state of knowledge on the role of particular chemokines and their receptors in the development and progression of AD. Special emphasis is given to their impact on forming Aβ and NFTs in humans and in transgenic murine models of AD.

## Background

### Prevalence, Symptoms, and Risk Factors for Alzheimer’s Disease

The prevalence of dementia around the world is rising at an alarming rate. According to the World Health Organization (WHO), more than 55 million people worldwide suffer from dementia. This number is estimated to nearly double by 2030 (78 million) and nearly triple by 2050 (139 million). The leading form of dementia is Alzheimer’s disease (AD), which along with other forms of dementia, is the seventh major cause of death globally, while in high-income countries it is ranked at the second place (WHO 2020, 2021).

Pathological hallmarks of the disease, i.e., senile plaques and neurofibrillary tangles (NFTs), appear in the third or fourth decade of life, approximately 15–20 years before the first symptoms of AD emerge (Ashford et al. 2011); this stage is defined as a preclinical AD. As the disease progresses, preclinical AD transits to mild cognitive impairment (MCI) in which cognition deficits do not affect patients’ performance at work or regular daily activities, but eventually may develop into AD (McKhann et al. 2011; Saido 2013). In 2011, the National Institute on Aging-Alzheimer’s Association (NIA AA) established the core criteria for diagnosis of probable AD–gradual onset of symptoms over months or years and unambiguous cognitive decline. An AD patient should present impairment in learning, especially affecting episodic memory, difficulties in word-finding, impaired spatial cognition, face recognition, alexia and object agnosia, impaired reasoning, judgment, and problem solving (McKhann et al. 2011). The earliest noticeable neuropsychiatric symptoms, occurring years prior to the diagnosis, include anxiety, depressive mood, apathy, and withdrawal (Atri 2019). Patients with advanced stage of AD may additionally become aggressive and agitated, experience sleep and appetite disturbances, delusions, and hallucinations (Zhao et al. 2016).

Age is considered to be a major risk factor for AD development. Indeed, majority of studies indicate that dementia occurs in individuals aged over 65 years and it is doubling with every 5 years of age. The prevalence of dementia rises up to 50% in those aged over 90 years. Thus, age of 65 years serves as an arbitrary borderline between two forms of AD, i.e., early-onset AD (EOAD, familial AD) and late-onset (LOAD, sporadic AD). In about half of the cases, EOAD has a hereditary origin associated with rare autosomal, dominant mutations of three genes encoding amyloid precursor protein (APP) and presenilin 1/2 (PSEN1, PSEN2) that activate γ-secretase. This form of AD is less frequent than LOAD, making up from 5 to 10% cases, but runs at a more aggressive course (Armstrong 2019; Ayodele et al. 2021; van der Flier et al. 2011). On the contrary, LOAD is related to a variety of factors, particularly senescence processes, metabolic diseases such as vascular disease or diabetes, traumatic brain injury, and dysfunction of the immune system (Armstrong 2019). Additionally, a carriage of apolipoprotein ε4 (APOE) allele is one of the strongest genetic risk factors for LOAD (Ayodele et al. 2021; Hampel et al. 2018; van der Flier et al. 2011).

### Anatomical and Molecular Changes in the Brain Induced by AD

As aging affects all systems of the body, age-related anatomical changes affect also the brain. In persons without symptoms of dementia, brain starts to decrease its weight at around 45–50 years old, primarily due to atrophy caused by a shrinkage of neurons and reduction of synapses rather than a neuronal loss. These alternations may impair to some extend working memory, executive functioning, and processing speed but the changes do not proceed fast. Moreover, linguistic abilities and verbal knowledge seems to be unaffected in normal aging processes (Sengoku 2020; Toepper 2017). In contrast, there are reports on AD documenting a profound loss of cholinergic neurons in the basal forebrain along with their axons projected to the cerebral cortex and limbic structures, including the amygdala and hippocampus, which leads to a subsequent denervation of critical regions responsible for memory, learning, cognition, neuroplasticity, and higher brain function (Hampel et al. 2018). Therefore, the brain affected by AD shows ventricle enlargement in the frontal and temporal cortices as well as atrophy of the gyri (Sengoku 2020).

Molecular changes in AD encompass formation and accumulation of pathological structures in the brain, namely extracellular senile plaques containing aggregates of amyloid β peptide (Aβ) and intracellular neurofibrillary tangles consisting of hyperphosphorylated tau protein (Saido 2013; Sengoku 2020). Amyloid precursor protein (APP) is a transmembrane glycoprotein presumably acting as a trophic factor involved in neuro- and synaptogenesis, neuronal migration and regeneration (Coronel et al. 2018). It can be metabolized in two distinct pathways: a dominant non-amyloidogenic and an amyloidogenic. In the main route, APP is cleaved by α-secretase (ADAM10; a disintegrin and metalloproteinase 10) within the Aβ domain, which results in releasing of a soluble ectodomain of APP, i.e., sAPPα. A remaining *C*-terminal fragment of the peptide (CTF-α, 83 aa) is then cleaved by γ-secretase to peptide P3 and APP intracellular domain (AICD) which is immediately degraded. In the amyloidogenic pathway, β-secretase cleaves APP to a soluble ectodomain of APP (sAPPβ) and a *C*-terminal fragment (CTF-β, 99 aa). Next, γ-secretase cleaves the CTF-β to Aβ and AICD. The latter can escape degradation when stabilized by an adaptor protein Fe65 and a histone acetyltransferase Tip 60. The complex interacts with the nucleus as a regulator of distinct genes expression, impeding neurogenesis, enhancing hyperphosphorylation catalyzed by glycogen synthase kinase 3β (GSK-3β) and aggregation of tau, but also regulating α-secretase activity. As γ-secretase cleaves CTF-β at various sites, Aβ is a heterogenous group of peptides, ranging from 38 to 49 amino acid residues in length (Brunholz et al. 2012; Coronel et al. 2018). Although Aβ40 constitutes approximately 90% of generated Aβ, the brain of AD patients contains mainly Aβ42. Aβ42 is strongly amyloidogenic and aggregates into dimers, oligomers, and lastly insoluble fibrils forming senile plaques, the hallmark of AD (Coronel et al. 2018).

The other neuropathological features of AD are NFTs. Tau protein belongs to microtubule-associated proteins (MAP) that shape cytoskeleton by regulating association and dissociation of microtubules and ensure proper neuronal transport (Jouanne et al. 2017). Tau is present in neurons, mainly in axons, although it can also be found in glial cells (Brunello et al. 2019). Under physiological conditions, more than 30 out of 80 serine and threonine residues of tau undergo phosphorylation; however, microtubules are preferably bound to a naïve tau form. Pathological tau arises as a result of deregulation of kinases and phosphatases action in processes of glycosylation, glycation, cleavage, nitration, ubiquitination, and mostly hyperphosphorylation. Due to the presence of additional phosphorylation sites, three- to fourfold increase in phosphorylation of tau protein can be found in patients with AD as compared to healthy individuals. Non-phosphorylated tau protein is a highly soluble monomer with a disorganized structure, whereas in AD, extensively phosphorylated tau (p-tau) changes its conformation in the misfolding process and loses the ability to bind to microtubules. Thus, p-tau level rises in cytosol and can be detected in cerebrospinal fluid (CSF). This increase is facilitated by insufficient tau degradation and leads to subsequent aggregation. Abnormally phosphorylated tau dimerizes, and then aggregates to soluble oligomers which are believed to be the most toxic species leading to progression of neurodegeneration. Finally, oligomers form paired helical filaments (PHFs) which build up to NFTs deposits in the brain (Brunello et al. 2019; Jouanne et al. 2017). Additionally, pathological tau can be secreted to extracellular space and enter surrounding neurons or glial cells further impairing axonal transport and causing neuronal synaptic loss thus contributing to progression of the disease (Brunello et al. 2019).

### Current Possibilities of Treatment of AD

Pharmacological therapy of AD is limited as there are only four commonly used drugs, i.e., donepezil, galantamine, and rivastigmine (second generation acetylcholinesterase inhibitors) or memantine, an NMDA receptor antagonist. Although they stabilize the patient’s condition, slow exacerbation of symptoms, or improve cognitive functions, they do not stop the progression of the disease (Hansen et al. 2008; Matsunaga et al. 2018). The aforementioned drugs were introduced to the market between 1997 and 2003. Since then there has been no other milestone in the treatment of AD (Jouanne et al. 2017). However, potential targets might be set thanks to recent studies providing strong evidence for the contribution of neuroinflammation to the pathophysiology of AD. The neuroinflammatory process may be ignited by infection, cellular debris, or by Aβ which activate microglia.

### The Role of Chemokines in the Brain

Chemokines are promising targets for anti-inflammatory drugs in AD. These small-molecule proteins orchestrate the neuroinflammation as they are involved in attraction and activation of microglia and may induce a leakage of the blood–brain-barrier (BBB) to allow infiltration of peripheral immune cells. Chemokines may be released by neurons, astrocytes, or microglia, and all these cell types can express certain chemokine receptors. Thus, chemokines play also an important role in cell-to-cell communication in the central nervous system (CNS).

Physiologically, microglia maintain proper functioning of neurons by regulating secretion of brain-derived neurotrophic factor (BDNF) and via phagocytosis of pathogens or cellular debris. However, in proinflammatory conditions, activated microglia impair synapse formation, neuronal plasticity, and facilitate inflammatory response releasing numerous cytokines, e.g., IL-1β, IL-6, TNFα, chemokines (i.e., CCL2, CCL4, CCL11), which attract further microglia and astrocytes to an inflammatory site (Minter et al. 2016). The recruited cells are not able to remove massive amounts of aggregated insoluble Aβ, so the cytokines are permanently synthesized leading to a subsequent dystrophia of microglia and degeneration of neurons which release their contents and damage-associated molecular patterns (Streit et al. 2018). The neuroinflammatory processes become unstoppable even if treated with anti-amyloid therapy (Minter et al. 2016), which calls into question whether the pathological cascade goes out of control despite removal of alleged primary stimulus or Aβ is only an intermediate product rather than a trigger. It is hypothesized that neuroinflammation occurs in the late stage of preclinical AD and determines its transition to symptomatic AD (Streit et al. 2018).

This review covers the current state of knowledge on the role of particular chemokines in the development of AD. Special emphasis is given to their impact on forming Aβ and NFTs in humans and in transgenic murine models of AD.

## Animal Models Used in Studies on AD Pathogenesis

In studies on the pathogenesis of AD, various transgenic (Tg) mouse models are used. They usually harbor human mutations responsible for familial Alzheimer’s disease (FAD), including variants of APP, presenilin 1 (PS1), and microtubule-associated protein tau (MAPT) (Hall and Roberson 2012; Puzzo et al. 2015). The most commonly used Tg murine AD models are listed below.

- The Tg2576 (also known as APP_Swe_ or R1.40) line, carrying the double Swedish mutation (K670N and M671L) at the β-secretase cleavage site. These mice display an increase of APP production (> fivefold) with consequent overproduction of Aβ40 and Aβ42, and plaques formation in the frontal, temporal, and entorhinal cortices, hippocampus, presubiculum, and cerebellum at about 11–13 months of age. Tg2576 mice can also display hyperphosphorylated tau at old age.
- The rTg4510 (JNPL3) line expressing human tau P301L.
- The APP/PS1 line containing human transgenes for APP (APP_*S*we_) and PS1 (L166P) mutation, both under control of the Thy1 promoter.

The hAPP/PS1 lines created by co-injecting the presenilin and hAPP transgenes:

- APP_Swe_/PS1ΔE9. It combines hAPP containing the APP_Swe_ and PS1 containing the ΔE9. APP_Swe_/PS1ΔE9 mice develop amyloid plaques and behavioral deficits around 6–7 months of age.
- The 3xTg, carrying Tg2576/APP_Swe_, P301L, and PS1 M146V mutations. 3xTg mice develop extracellular Aβ plaques before tangle pathology, as in human AD.
- The 5xFAD, created by combining five AD-related mutations–three mutations in hAPP: Swedish (K670N and M671L), Florida (I716V), and London (V717I), and two mutations in PS1: M146L and L286V. In 5xFAD mice, rapid amyloid pathology was observed as early as at 2 months of age. These animals lose 25–40% of layer 5 pyramidal neurons between 9 and 12 months of age.

## Chemokines and Chemokine Receptors

Chemokines are a large family of small (8–14 kDa) structurally and functionally related proteins, subdivided into four groups on the basis of a relative position of two *N*-terminal residues of four conserved cysteines. One and three amino acids separate the first and second cysteines in the CXC and CX3C, respectively, whereas two cysteines are adjacent to each other in the CC subfamily. CXC (or α-chemokines) and CC (or β-chemokines) are the largest groups, while CX3C (or δ-chemokine) includes only one member, CX3CL1/fractalkine, in which the first two cysteines are separated by three amino acids. This chemokine exists in both soluble and membrane-bound forms (Bajetto et al. 2002; Bazan et al. 1997; Pan et al. 1997). The best-known function of chemokines is regulation of migration of various cells in the body, hence their name (from “chemotactic cytokines”). Numerous data, accumulated mostly during the last two decades, demonstrate that chemokines play a fundamental role not only in the development, homeostasis, and function of the immune system, but are also important for angiogenesis, cancer (in particular in terms of metastasis regulation), and the functioning of CNS. CNS produces chemokines, such as CX3CL1, CCL2, CXCL8, CXCL10, CCL5, and CCL3 (also known as macrophage inflammatory protein 1-α; MIP-1 α) (Hughes and Nibbs 2018).

**Fig. 1:**
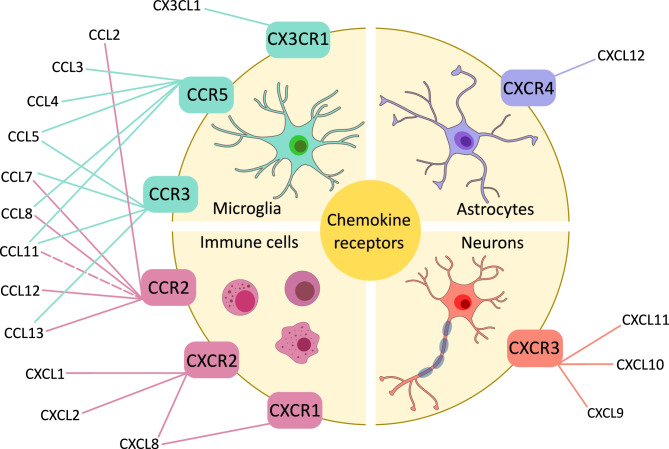
Schematic representation of chemokines and their receptors

Chemokines exert their biological activity by binding to cell surface receptors that belong to the superfamily of G protein-coupled receptors. Although multiple chemokines can often bind to the same receptor and a single chemokine can bind to several receptors (see Fig. 1), the chemokine–chemokine receptor interactions are almost always restricted within a single subclass. To date, six CXC receptors have been identified, named from CXCR1 to CXCR6, and eleven CC receptors, from CCR1 to CCR11, along with a single receptor for CX3CL1 and one for lymphotactin α/β, called CX3CR1 and XCR1, respectively (Hughes and Nibbs 2018).

As neuroinflammation plays a significant role in pathogenesis of AD, it is crucial to understand in detail the signaling pathways related to it. In recent years, numerous studies have been conducted in order to elucidate the relationship of chemokine signaling with accumulation of Aβ and hyperphosphorylated tau, leading to neurodegeneration and dementia. Such knowledge may in future give foundations for development of novel class of disease-modifying treatments for AD.

### CX3CL1 (Fractalkine)

CX3CL1 (fractalkine) is a chemokine constitutively expressed by neurons under physiological conditions. Unlike many other chemokines, fractalkine interacts with only one receptor, CX3CR1, expressed on microglia, thus playing a vital role in neuron-microglia communication (Rogers et al. 2011). Its main physiological role is to maintain a resting state of microglia. Decreased CX3CL1 signaling results in microglial activation into a pro-inflammatory state, and phagocytosis of synaptic elements (Bisht et al. 2016; Bolós et al. 2017; Rogers et al. 2011; Zhang et al. 2018). Apart from its impact on microglia, CX3CL1 plays a crucial role in neurogenesis. It promotes proliferation of neural stem cells and inhibits their differentiation into astrocytes. Overexpression of this chemokine leads to increased adult neurogenesis in the subgranular zone of the hippocampus (Fan et al. 2020; Shang et al. 2019). On the other hand, disruption of CX3CL1 signaling inhibits hippocampal neurogenesis in rodents by decreasing proliferation and survival of neural progenitor cells. In aged rats, a decrease of hippocampal neurogenesis is paralleled by reduction of CX3CL1 levels, while treatment with exogenous chemokine reverses this process (Bachstetter et al. 2011). Importantly, disturbed neurogenesis in CX3CR1-deficient mice is manifested by a cognitive dysfunction, impaired motor learning, and reduced long-term potentiation (LTP), reflecting reduction of synaptic plasticity mediated by the increased IL-1β signaling (Rogers et al. 2011).

The hippocampus, the brain structure responsible for various cognitive functions that depend on adult neurogenesis and synaptic plasticity, is one of the earliest affected brain regions in AD, and its dysfunction is believed to underlie the core feature of the disease-associated memory impairment (Rao et al. 2022). Reduced levels of CX3CL1 were found in the hippocampus and frontal cortex of AD patients as compared to healthy controls (Cho et al. 2011), and in the CSF of patients with MCI and AD dementia (Perea et al. 2018). Furthermore, in AD patients, hippocampal CX3CL1 mRNA levels were markedly lower at the late stage of the disease (Braak-Tau stages V-VI) as compared to the intermediate one (Strobel et al. 2015). It is postulated that reduction of CX3CL1 levels results from a decline of viable neurons in patients with severe AD (Strobel et al. 2015). Conversely, other reports demonstrated increased levels of CX3CL1 protein in the hippocampi of early (Braak-Tau stages II-IV) (Lastres-Becker et al. 2014) and late stage of AD (Braak-Tau stages V-VI) (Dworzak et al. 2015) as compared to healthy controls. Interestingly, it was shown that tau competes with CX3CL1 for binding to its receptor (Chidambaram et al. 2020), an observation suggesting that upregulation of this axis may play a compensatory role (Bolós et al. 2017).

Involvement of the CX3CL1/CX3CR1 axis in the pathogenesis of AD is also well-documented in animal models. Cultured wild-type mouse neurons secreted soluble form of CX3CL1 prior to death in response to Aβ (Dworzak et al. 2015). CX3CL1 release from cultured mouse microglia and neurons is also increased in response to tau. In line with these in vitro observations, neurons injured by a stereotactical delivery of tau into the mouse hippocampus expressed elevated levels of CX3CL1 (Lastres-Becker et al. 2014). Furthermore, Aβ injection into the CA1 region of the rat hippocampus resulted in increased expression and protein level of CX3CR1. Interestingly, genetic ablation of CX3CR1 abolished microglial activation, neuroinflammation, and cognitive deficits in Aβ-treated rats (Wu et al. 2013). On the other hand, decreased expression of CX3CL1 was found in the cerebral cortex of Tg2576 mice at 9 and 17 months of age (Duan et al. 2008).

Studies utilizing Tg murine models of AD provided intriguing data on effects of manipulations of the CX3CL1/CX3CR1 axis on outcomes of two hallmarks of the disease, i.e., Aβ deposition and formation of NFTs. Inhibition of CX3CL1 signaling produces opposite effects on Aβ deposition and tau phosphorylation in various Tg mouse lines. CX3CL1-deficient APP/PS1 mice exhibit enhanced phosphorylation of neuronal tau and reduced Aβ accumulation (Lee et al. 2014). Similarly, knock-out of CX3CR1 in APP mice resulted in exacerbated tau pathology, cognitive decline, and memory loss, paralleled by increased levels of the pro-inflammatory cytokine IL-6 (Cho et al. 2011). Also, in Tg mice expressing human tau, lack of CX3CR1 resulted in enhancement of tau phosphorylation and aggregation, a process dependent on activation of p38 MAPK and microglial activation. Of note, these animals exhibited pronounced behavioral impairments (Bhaskar et al. 2010). The worsening of tau pathology in CX3CR1-deficient mice may be mediated by a reduced capacity of microglia to phagocytose the protein. Indeed, it has been shown that due to a structural similarity to CX3CL1, tau can bind to CX3CR1 leading to its internalization by microglia expressing this receptor (Bolós et al. 2017). Similarly to changes observed in mice with disrupted CX3CL1 signaling, overexpression of this chemokine in neurons of mice harboring human tau mutation prevents neurodegeneration, improves cognitive functions, and extends lifespan. However, this effect did not involve reduced microglial activation (Fan et al. 2020).

On the other hand, disruption of the CX3CL1/CX3CR1 axis ameliorates Aβ burden in various Tg murine models of AD. APP/PS1 mice heterozygous for CX3CR1 had reduced Aβ levels as compared to age-matched APP/PS1. This was paralleled by enhanced expression of Aβ-clearing enzymes in neurons, and resulted in cognitive improvement (Hickman et al. 2019). A similar reduction in Aβ deposition accompanied by reduced microglial activation and accumulation of microglia around Aβ deposits was observed in CX3CR1-deficient APP/PS1 mice and in APP_Swe_ mice. Noteworthy, the reduction of Aβ deposition was not mediated by changes in APP processing but by increased microglial phagocytosis (Lee et al. 2010). In a different mouse model, CRND8 harboring a human mutation of APP (APP_Swe_ and APP V717F), knock-out of CX3CR1 reduction of Aβ deposits mediated by increased microglial uptake was also demonstrated. However, in this model, the number of microglial cells surrounding Aβ plaques was increased (Liu et al. 2010b). This discrepancy may result from a different genetic background and modifications between both Tg strains. On the other hand, knock-out of CX3CR1 in a 3xTg mice prevented neuronal loss, but not affected Aβ deposition and phagocytosis by microglia (Fuhrmann et al. 2010).

CX3CL1, which is primarily expressed in its membrane-bound form, can be cleaved by ADAM10/ADAM17 and secretases, giving rise to soluble CX3CL1 (sCX3CL1, build-up of chemokine domain and mucin stalk) and membrane-anchored fragments of the peptide (Bemiller et al. 2018; Fan et al. 2019). Importantly, different effects on CX3CL1 forms have been observed. For instance, overexpression of the *C*-terminal membrane fragment of CX3CL1 in 5xFAD mice led to reduction of Aβ deposition due to decreased APP expression and spared neuronal loss resulting from enhanced neurogenesis. Those effects were independent of CX3CR1, and mediated by a direct translocation of *C*-terminal fragment into the nucleus, leading to changes in gene expression (Fan et al. 2019). Furthermore, lack of the membrane-anchored CX3CL1 in AD mice expressing only the soluble chemokine domain of the peptide (CX3CL1^105Δ^) resulted in increased expression of proinflammatory markers, including cytokines, reduction of CX3CR1 expression in microglia, and hyperphosphorylation of tau; these changes were accompanied by deficits in spatial learning (Bemiller et al. 2018; Lee et al. 2014). It is suggested that the bidirectional effects of CX3CL1 deficiency on the Aβ and tau pathologies in APP/PS1 mice may depend on the absence of the membrane-bound fragment, as overexpression of sCX3CL1 form did not abolish them (Lee et al. 2014).

In rTg4510 mice, overexpression of sCX3CL1 using an adeno-associated viral vector in young animals, prior to a significant tau accumulation, resulted in reduction of tau pathology, microglial activation, and neurodegeneration, without cognitive improvement. However, when injected using the same vector to older rTG4510 mice, with tau pathology reaching its plateau, sCX3CL1 produced cognitive improvement, without reversing tau hyperphosphorylation or damage of the hippocampus (Finneran et al. 2019; Nash et al. 2013). Amelioration of cognitive impairment in AD mice by sCX3CL1 is additionally supported by findings that treatment with this form of the chemokine in CX3CL1^−/−^ non-AD mice restores hippocampal neurogenesis and synaptic plasticity, being reflected in improvement of cognitive functions and memory (Winter et al. 2020). On the other hand, overexpression of sCX3CL1 did not change Aβ pathology in APP/PS1 mice (Nash et al. 2013). These contrasting effects may be related to significantly increased CX3CR1 expression in rTg4510 mice, while levels of sCX3CL1 in APP/PS1 did not differ from wild-type littermates. Therefore, physiological levels of CX3CL1 may exert full effects in APP/PS1 mice, whereas in rTg4510 mice CX3CL1 signaling may by further stimulated by the additional soluble form of this chemokine (Nash et al. 2013). However, it is possible that the effects of CX3CL1 on the Aβ burden in APP/PS1 mice are mediated mainly by membrane-bound fragment of this chemokine, as reported elsewhere, or that the presence of mucin stalk in CX3CL1 fragment is necessary to produce neuroprotective effects (Fan et al. 2019; Lee et al. 2014).

#### CCL2 (MCP-1)

CCR2 receptors are expressed on immune cells and can be activated by pro-inflammatory chemokines, including CCL2 (also known as monocyte chemoattractant protein-1, MCP-1), CCL7, CCL8, CCL12, and CCL13. Among them, CCL2 is of particular interest, as it is the most potent activator of CCR2 (Chu et al. 2014). In the CNS, CCL2 induces migration and accumulation of peripheral immune cells and microglia into an endangered/damaged site. It is also involved in the neuroinflammatory reaction by promoting transition of microglia from resting into activated phenotype. Additionally, CCL2 was found to promote the loss of the BBB integrity by disruption of adherent junctions, thus facilitating infiltration of peripheral immune cells into the CNS. In normal conditions, neuroinflammation plays a neuroprotective role against various harmful factors. However, when microglia and astrocytes fail to clear toxic forms of Aβ, chronic elevation of chemokines levels, resulting in prolonged activation of glial cells and neuroinflammation, may contribute to neuronal loss and a disease progression (Kiyota et al. 2009a; Li et al. 2009; Nordengen et al. 2019; Rezazadeh et al. 2016; Roberts et al. 2012; Sui et al. 2019).

There is accumulating evidence for involvement of the CCL2/CCR2 axis in pathogenesis of AD. Elevated levels of CCL2 were found in plasma and CSF (Corrêa et al. 2011; Galimberti et al. 2006; Lee et al. 2018; Nordengen et al. 2019; Stuart and Baune 2014), and brain tissue, including critical structures for AD, such as the hippocampus, frontal, and temporal cortex of AD patients as compared to healthy controls (Liao et al. 2016; Sokolova et al. 2009). In some of these studies, the highest levels of CCL2 were observed in patients with severe AD, and they correlated with progression of cognitive decline or CSF concentration of Aβ and hyperphosphorylated tau (Corrêa et al. 2011; Lee et al. 2018). It is postulated that high expression of CCL2 and CCR2 are a risk factor for AD development (Murcia et al. 2020; Rezazadeh et al. 2016). High levels of CCL2 in patients with prodromal AD were associated with a faster cognitive decline (Westin et al. 2012). Importantly, it has been found that Aβ stimulates CCL2 release from astrocytes and microglia in vitro (El Khoury et al. 2003; Ito et al. 2006; Kiyota et al. 2013; Smits et al. 2002). Additionally, CCL2 is present in mature senile plaques and reactive microglia, where it is involved in microglial accumulation and phagocytosis of Aβ (Ishizuka et al. 1997; Sui et al. 2019). Similarly to humans, changes in expression of CCL2 were observed in murine models of AD. Increased expression of this chemokine was observed in brains of female 5xFAD at 5 months of age, i.e., at a stage of significant Aβ deposition (Manji et al. 2019). Likewise, increased levels of CCL2 were found in brains of aged mice representing other Tg lines, such as Tg2576, 3xTg AD, and APP/PS1 (Hartlage-Rübsamen et al. 2015; Reale et al. 2018; Zaheer et al. 2013). In the APP/PS1 model, the cerebral expression of CCR2 was also elevated (Krauthausen et al. 2015).

Although overexpression of CCL2 in mice does not directly alter cognitive functions (Kiyota et al. 2009b), its impact on the symptoms and progression of AD has been widely studied using Tg animals. In APP mice, overexpression of CCL2 results in increased deposition of Aβ in the cerebral cortex and hippocampus, coinciding with enhanced accumulation of mononuclear phagocytes around senile plaques, and leading to cognitive decline. Interestingly, accumulation of Aβ in CCL2 overexpressing mice was neither mediated by alternations of APP expression or processing, nor by suppression of Aβ degradation, but resulted from stimulation of oligomerization of Aβ peptides giving rise to an insoluble, fibrillar form of the amyloid. It has been demonstrated that increased levels of CCL2 in APP mice evoke enhanced microglial accumulation, activation, and phagocytosis of Aβ monomers which are then conversed intracellularly into oligomers. Secreted Aβ oligomeric species act as seeds accelerating diffuse plaque formation, thus contributing to progression of the disease (Kiyota et al. 2009b; Yamamoto et al. 2005). Similarly, upregulation of CCL2 produces detrimental effects in rTg4510 mice, where it caused microglial activation and aggravated tau pathology by increasing the insoluble tau fraction, leading to extended generation of NFTs (Joly-Amado et al. 2020).

Aside from overexpression of CCL2, also silencing of the CCL2/CCR2 axis can lead to aggravation of AD pathology in murine models. In APP/PS1 mice, knock-out of CCR2 accelerates accumulation of intracellular soluble Aβ oligomers that was paralleled with decline of cognitive functions. Surprisingly, increased recruitment of microglia around senile plaques was observed in the prefrontal cortex and hippocampus of APP/PS1/CCR2^−/−^ mice. This finding suggests involvement of a compensatory mechanism leading to migration of microglia. Microglia of APP/PS1/CCR2^−/−^ mice exhibit increased expression of CX3CR1 which is involved in inhibition of microglial activation and phagocytosis (Naert and Rivest 2011). A deficiency of CCR2 leads to acceleration of AD progression also in Tg2576 mice, as accumulation of Aβ occurs faster due to decreased clearance by microglia, and results in a premature death of animals (El Khoury et al. 2007). Similarly, CCR2 deficiency in bone marrow cells in APP/PS1 mice also led to aggravation of cognitive impairment and increased levels of soluble Aβ oligomers, pointing to the role of bone marrow-derived CCR2-positive microglia in phagocytosis of soluble Aβ (Naert and Rivest 2012). Deficiency of CCL2 in APP/PS1 mice is also involved in behavioral impairment and increased levels of soluble Aβ oligomers in the hippocampus, a process mediated by impairment of microglial phagocytosis, despite its accumulation around compact plaques (Kiyota et al. 2013]. Furthermore, in Tg2576 mice, knock-out of CCR2 resulted in decreased clearance of Aβ by perivascular macrophages and accumulation of insoluble Aβ species (Mildner et al. 2011). Knock-out of CCL2 in APP/PS1 mice led to impaired neurogenesis; this was not observed in non-Tg CCL2^−/−^ mice. Therefore, it can be concluded that the interaction of familial AD mutations and CCL2 deficiency results in impaired neuronal proliferation and differentiation, which can further contribute to the disease progression (Kiyota et al. 2013). Similarly, in mice harboring PS1 mutation, deficiency of CCL2 led to impairment of adult hippocampal neurogenesis and synaptic plasticity, manifested as deficits in learning and memory (Kiyota et al. 2015). This observation is in line with findings obtained with APP/PS1 mice (Kiyota et al. 2013), and supports the role of the CCL2/CCR2 axis not only in chemotaxis, but also in architecture of the CNS.

On the other hand, suppression of CCL2 by administration of a dominant-negative CCL2 mutant gene *7ND* into the hippocampus of APP/PS1 mice at the pre-symptomatic stage using the adeno-associated virus resulted in attenuation of microgliosis and decreased formation of oligomeric and fibrillary Aβ (Kiyota et al. 2009a); this observation is analogous to results obtained in experiments using CCL2 overexpression (Kiyota et al. 2009b). Interestingly, when administered to APP/PS1 mice at the post-symptomatic stage, *7ND* produced cognitive improvement (Kiyota et al. 2009a). In 5xFAD mice, knock-out of CCL2 reduced neuroinflammation, accumulation of senile plaques, and neuronal loss leading to alleviation of behavioral deficits observed at the post-symptomatic stage of the disease (Gutiérrez et al. 2019a, b).

Summarizing the above observations, it seems that the CCL2/CCR2 axis, when functioning at the physiological level, plays a protective role against amyloidosis by increasing microglial accumulation and clearance of Aβ, especially at the early stage of pathology. On the other hand, at the late stage of AD, CCL2/CCR2 may aggravate the pathology by stimulation of excessive neuroinflammation and promotion of insoluble Aβ formation inside microglial cells.

#### CCL3, CCL4, CCL5

Although there are published data on the relationship between CCR5 and its ligands with AD, this area is explored to a lesser extent than CX3CL1/CX3CR1 or CCL2/CCR2. CCR5 is expressed on microglia in the CNS and interacts with several chemokines, such as macrophage inflammatory proteins 1α and 1β (CCL3 and CCL4, respectively), RANTES (regulated on activation, normal T cell expressed and secreted, CCL5), CCL8, and eotaxin (CCL11) (Necula et al. 2020; Ogilvie et al. 2001). Agonists of CCR5 possess chemoattractant properties, promote infiltration of immune cells, their accumulation, and pro-inflammatory action (Laurent et al. 2017; Li et al. 2009; Skuljec et al. 2011).

Expression of CCR5 and its ligands in the CNS and peripheral blood of AD patients differs from control subjects. Levels of CCL5 mRNA in peripheral blood were found to be lower in AD patients as compared to age matched controls (Kester et al. 2011). However, in this study, the sample size was relatively small (*n* = 23 for each group), and the subjects with subjective complaints but not fulfilling the criteria for mild cognitive impairment constituted the control group. In another study, levels of CCL3 in peripheral blood of AD patients were lower as compared to those observed in controls, but did not correlate with the severity of dementia (Geppert et al. 2010). Noteworthy, in this study, patients with other neurological disorders served as a control group; therefore, it does not provide information on whether CCL3 levels in plasma of AD patients differ from healthy humans.

Elevated levels of CCL5 were found in plasma and brain microvessels of AD patients as compared to controls, with a negative correlation between CCL5 level and age as well as duration of the disease. However, levels of CCL5 did not correlate with cognitive scores (Tripathy et al. 2010; Vacinova et al. 2021). Elevated expression of CCR5 was observed in lymphocytes T and B of AD patients as compared to healthy controls. Interestingly, treatment with a recombinant Aβ resulted in increased surface expression of CCR5 on both types of lymphocytes isolated from AD patients (Pellicanò et al. 2010). CCR5 is present on microglia of AD patients and controls; its increased expression was observed in reactive microglia of AD patients. Additionally, CCL4 was found predominantly in reactive astrocytes, which were more widespread in AD brains. CCR5-positive microglia and CCL4-positive astrocytes were associated with Aβ deposits (Bakshi et al. 2011). Similarly, in AD patients, increased expression of CCL3 was found in neurons of the hippocampus, temporal, and frontal regions of brains. Treatment with Aβ induced increased expression of CCL3 in a model human SH-SY5Y cell line derived from neuroblastoma (Liao et al. 2016).

A significant relationship between apolipoprotein E (APOE) polymorphism and CCR5 ligands has been reported in brains of AD patients. APOE is involved in cholesterol homeostasis and immune response in the CNS. Its allele ε4 encoding apoE4 isoform is a major risk factor for sporadic form of AD. It has been found that stimulated humanized murine ε4 astrocytes secrete more CCL3 than ε3 astrocytes, and the level of CCL3 in brains of AD patients homozygous for APOE ε4 is significantly higher as compared to age-matched homozygous APOE ε3 AD individuals (Cudaback et al. 2015).

Although expression of CCR5 ligands is altered in AD patients, numerous studies report that CCR5^Δ32^ mutation, resulting in the loss of CCR5 functionality, does not protect from AD, as the frequency of this allele does not differ between AD patients and healthy controls (Balistreri et al. 2006; Combarros et al. 2004; Huerta et al. 2004; Wojta et al. 2020). It was noted, however, that patients carrying CCR5^Δ32^ mutation develop AD at a younger age (Wojta et al. 2020).

Treatment with Aβ1-42 resulted in increased expression of CCL3 and CCL4 in cultured murine microglia (El Khoury et al. 2003; Ito et al. 2006), and in cortical human microglia isolated from post-mortem cases (Walker et al. 2001). Moreover, Aβ induced mRNA expression of CCR5 in THP-1 monocytes (human cell line used as a model of microglia) and in human peripheral blood monocytes, which in turn stimulated chemotaxis of monocytes toward CCL4 and CCL5 gradient (Giri et al. 2005). Upregulation of CCR5 was also stimulated by Aβ in human brain microvascular endothelial cells which constitute the BBB (Li et al. 2009), while in rats intrahippocampal injection of Aβ resulted in overexpression of CCR5 in brain endothelial cells, and CCL3 in peripheral T lymphocytes (Li et al. 2009; Man et al. 2007). In wild-type mice, intracerebroventricular injection of Aβ1-40 resulted in increased expression of CCL3, followed by astrocytosis, microgliosis, and neuroinflammation, finally leading to cognitive impairment. All these negative effects were reduced in CCL3^−/−^ and CCR5^−/−^ mice (Passos et al. 2009). The observed upregulation of CCR5 and its ligands are of significant importance as they aggravate neuroinflammatory reaction by promoting accumulation of resident microglia and infiltration of T cells and peripheral monocytes through the BBB into the brain, where the latter can undergo differentiation into microglia (Giri et al. 2005; Li et al. 2009; Man et al. 2007; Passos et al. 2009).

Studies utilizing Tg murine models shed more light on involvement of CCR5 and its ligands in the pathogenesis of AD. In the double transgenic APP/PS1 model of amyloidosis, increased levels of CCL3 and CCL4 were found in the brain within a frame of 7 to 15 months of age (Jorda et al. 2019; Martin et al. 2019; Zhu et al. 2014). CCL3 was present in astrocytes and microglia (Martin et al. 2017), while CCL4 was predominantly expressed by reactive astrocytes associated with Aβ deposits (Zhu et al. 2014). At 7 months of age, expression of CCR5 decreased, while that of CCL5 remained unchanged (Jorda et al. 2019). However, other studies using older mice reported no changes in CCR5 expression (brains dissected at various ages; median of 10.9 months) (Zhu et al. 2014) or increased expression of CCL5 at 10 and 15 months of age (Martin et al. 2019). Additionally, expression of CCL4 directly correlated with progression of Aβ accumulation in the brain (Zhu et al. 2014). Therefore, it can be assumed that CCR5 axis is generally enhanced in this murine Tg AD model, and this overexpression progresses along with the pathology of the disease. Overexpression of CCL3, CCL4, and CCL5, paralleled by infiltration of T lymphocytes into the hippocampus, was also observed in a Tg murine model of tauopathy, THY-Tau22 (Laurent et al. 2017). Chronic infusion of lipopolysaccharide (LPS) into the fourth ventricle of young rats evoked astrogliosis and microgliosis in the hippocampus. These pathological changes were dramatically reduced by administration of CCR5 antagonist, D-Ala-peptide T-amide (DAPTA) (0.01 mg/kg, s.c., for 14 days) (Rosi et al. 2005). Of note, a decreased function of CCR5 in non-AD mice resulted in improvement of LTP and spatial memory, while its overexpression resulted in memory deficits (Zhou et al. 2016). Similarly, in non-AD mice, subchronic intracerebroventricular injection of CCL3 resulted in inhibition of LTP and cognitive deficits, processes mediated by CCR5, as its antagonist abolished these effects (Marciniak et al. 2015).

On the other hand, transplantation of bone marrow mesenchymal stem cells (BM-MSCs) into the hippocampus of APP/PS1 mice at 30 weeks of age resulted in increased expression of CCL5 mRNA as compared to sham-transplanted animals. However, in these mice, an alternative activation pattern of microglia was observed. It led to decreased Aβ deposition and improved cognitive performance (Lee et al. 2012). In experiments done on non-Tg mice, CCR5 deficiency led to compensatory overexpression of CCR2 which was associated with impairment of spatial memory, increased activity of β-secretase, and increased Aβ deposition (Lee et al. 2009). Accordingly, knock-out of CCR5 associated with elevation of CCL2 and CCR2 expression in the brain, resulted in higher susceptibility of mice to LPS treatment, manifesting in more pronounced cognitive impairment, activation of astrocytes, expression of inflammatory enzymes and β-secretase, and Aβ deposition as compared to CCR5 wild-type mice (Hwang et al. 2016). The findings presented above suggest that CCR5 ligands may play a protective or negative role in AD pathology, depending on the context. CCR5 is involved in immune response in AD brains, which is beneficial at the initial stage of the response; however, under chronic conditions, effects of stimulation of these receptors are detrimental. On the other hand, permanent silencing of CCR5, as in CCR5^−/−^ mice, leads to the development of compensatory upregulation of CCR2 and its ligand CCL2, resulting in aggravation of neuroinflammation and disease pathology.

#### CXCL8 (IL-8), CXCL2, CXCL1

Substantial evidence points to an association between disturbances of CXCL8 signaling and the pathomechanism of AD. Injection of Aβ1-42 into the rat hippocampus resulted in enhanced expression of CXCR2 and CXCL8, paralleled by significant gliosis and increased presence of T lymphocytes in the brain. In addition, SB33223 (a competitive CXCR2 antagonist) reduced microgliosis, accumulation of T lymphocytes and oxidative stress, leading to alleviation of neuronal loss in the dentate gyrus of Aβ-injected rats (Ryu et al. 2015; Liu et al. 2010a). Similarly, treatment of APP_Swe_ mice with SB225002, a selective CXCR2 antagonist, reduced soluble Aβ40 brain levels relative to the control group (Bakshi et al. 2008, 2011). Experiments performed on cell lines revealed that SB225002 lowered Aβ40 and Aβ42 levels. As SB225002 decreased γ-secretase activity via reduction of presenilin expression, it is suggested that effects of this CXCR2 antagonist resulted from its interference with the production of Aβ but not its secretion (Bakshi et al. 2008, 2011). Levels of CXCL8 were found to be significantly higher in samples, such as brain tissue, plasma, and CSF, obtained from AD patients as compared to healthy controls (Alsadany et al. 2013; Ashutosh et al. 2011; Corrêa et al. 2011; Galimberti et al. 2006; Sokolova et al. 2009). In brains of AD patients, elevated levels of CXCL8 were detected in neurons and around Aβ plaques (Sokolova et al. 2009). CXCR2 was also upregulated in peripheral T lymphocytes of AD patients, facilitating their entry into the CNS through the BBB (Liu et al. 2010a). Notably, in AD patients, levels of CXCL8 were negatively correlated with the cognitive score (Alsadany et al. 2013), and positively correlated with CSF Aβ levels (Corrêa et al. 2011). In vitro studies demonstrated a protective role of CXCL8 against Aβ-induced neurotoxicity. Human fetal microglia, astrocytes, and neurons stimulated with Aβ produced high amounts of CXCL8. Although CXCL8 alone did not alter neuronal survival, it did inhibit Aβ-induced neuronal apoptosis and increased production of BDNF (Ashutosh et al. 2011). On the other hand, treatment of primary neuronal rat cultures with CXCL8 in the absence of Aβ resulted in cell death and increased expression of pro-inflammatory matrix metalloproteinases 2 and 9, along with pro-apoptotic proteins (Thirumangalakudi et al. 2007). A recent meta-analysis indicates that a polymorphism (− 251 T > A) of the *CXCL8* gene is associated with increased risk of developing AD (Qin et al. 2016). In agreement with the clinical data, in vitro treatment with Aβ increased expression of various inflammation-related genes in cortical human microglia isolated from post-mortem cases, among which, expression of *CXCL8* was the most highly elevated (11.7-fold at the mRNA level) (Walker et al. 2001). CXCR2, the main receptor for CXCL8, is strongly upregulated in AD brains, where it is localized mainly on microglia around senile plaques (Ryu et al. 2015; Xia et al. 1997).

Apart from CXCL8, other ligands of CXCR2 are related with AD. Protection of hippocampal neurons against Aβ was observed for CXCL1 (also known as GROα*/*KC) and CXCL2 (Watson and Fan 2005). CXCL1 acts as a growth factor by stimulating neuronal ERK1/2 and PI-3 kinase pathways in cultured mouse neurons. However, this chemokine increases tau phosphorylation, which may limit its neuroprotective activity (Xia and Hyman 2002). Similarly, CXCL1 was found to induce phosphorylation and subsequent caspase-3 dependent truncation of tau protein, leading to formation of varicosities or bead-like structures along the neuritis. Tau cleavage, which is considered an early event in AD development, was also observed after intrahippocampal microinjection of lentiviral CXCL1 in aged (15–18 months of age), but not in adult (5–10 months of age) mice (Zhang et al. 2015). Pretreatment of mouse neurons with CXCL2 abolished Aβ-induced oxidative stress, dendritic regression, and neuronal apoptosis. This effect was independent of CXCR2, but attributed to CXCR1, as it was observed in cultured neurons from both CXCR2^+/+^ and CXCR2^−/−^ mice. Of note, CXCL2^−/−^ mice displayed significantly elevated expression of CXCR1 (Raman et al. 2011).

Monocytes obtained from AD patients overexpress CXCL1. Treatment of human brain microvascular endothelial cells with Aβ results in increased expression of CXCR2 which, via interaction with CXCL1, is involved in the breakdown of the BBB integrity and increased migration of monocytes into the brain of AD patients (Zhang et al. 2013). Similarly, levels of CXCL1 and CXCL2 were elevated in brains of APP/PS1 mice as compared to wild-type animals (Martin et al. 2019; Watson and Fan 2005), and treatment of mouse microglia with Aβ1-41 induced expression of CXCL2 (Ito et al. 2006).

#### CXCL12/CXCR4

CXCL12, also known as stromal cell-derived factor 1 (SDF-1), is a homeostatic chemokine expressed under physiological conditions at various sites, including the CNS, where it is produced mainly by astrocytes (Parachikova and Cotman 2007). CXCL12, by stimulating the CXCR4 receptor, promotes neurogenesis and synaptic plasticity, both during development and in adulthood. CXCL12-dependent adult neurogenesis and synaptic plasticity in the dentate gyrus is involved in learning and memory, as wild-type mice treated with a CXCR4 antagonist show cognitive impairment (Parachikova and Cotman 2007).

So far, the available data on the relationship between disturbances of CXCL12/CXCR4 axis and AD are scarce. First studies on this subject reported decreased levels of CXCL12 in plasma of early-stage AD patients. They were negatively correlated with those of tau protein in the CSF, but positively correlated with cognitive decline. This observation supports the idea of the relationship between deficiency of regenerative effects of CXCL12 with AD pathology (Laske et al. 2008). A study done on a large population of subjects–2139 healthy, non-demented persons (NDHC) and 1170 AD patients–demonstrated that brains of AD patients expressed high levels of CXCR4 and CHI3L1 (chitinase-3 like-protein-1 involved, among others, in inflammation, tissue injury and repair, remodeling, and firstly identified as a potential candidate for CSF biomarker [Zhao et al. 2020]), and low levels of CXCL12 and neurogranin (a protein playing an important role in synaptic plasticity, synaptic regeneration, and long-term potentiation, and a biomarker of neurological and mental diseases) (Xiang et al. 2020) as compared to NDHC subjects (Sanfilippo et al. 2020).

Similarly to AD patients, expression of *CXCR4* gene is upregulated in brains of rTg4510 murine model of tauopathy (Bonham et al. 2018) and APP/PS1 model of amyloidosis (Krauthausen et al. 2015). rTg4510 mice develop NFTs pathology in the hippocampus and neocortical regions by 6 months of age, while the cerebellum fails to these pathological changes. The increased expression of *CXCR4* in rTg4510 mice was detected in the hippocampus, a site of NFTs occurrence, but not in the cerebellum, where NFTs are not generated. Additionally, elevated *CXCR4* in the hippocampus co-localized with markers of microglial activation (*TMEM119* and *AIF1*, encoding transmembrane protein 119 and allograft inflammatory factor 1, respectively); this was associated with changes in expression of other microglia-related genes (upregulation of *TLR2* and down-regulation of *CCR5*) (Bonham et al. 2018). Alternations in the CXCL12/CXCR4 axis were also observed in Tg2576 mice harboring the human APP mutation. The hippocampal expression of both CXCL12 and CXCR4 were decreased in Tg2576 mice at 6 and 12 months of age as compared to age-matched non-transgenic animals; this was paralleled with cognitive impairment (Parachikova and Cotman 2007). Conversely, in wild-type C57BL/6 mice injected with Aβ, expression of CXCL12 was increased in injected regions. Similar results were observed in Tg2576 mice at 12 months of age (Wu et al. 2017). It should be noted that at this stage, the Tg2576 mice exhibit higher levels of soluble and insoluble Aβ, with the latter increasing 10,000-fold between these time points (Parachikova and Cotman 2007). Therefore, it can be assumed that expression of CXCL12 in the brain of Tg2576 (APP_Swe_) mice changes over time, reflecting the progression of Aβ pathology. In the case of a massive accumulation of Aβ or its rapid emergence due to injection, upregulation of CXCL12 may be related to a compensatory reaction. The protective action of CXCL12 against AD pathology was observed using Tg murine models. In APP/PS1 mice, intracerebroventicular administration of CXCL12 resulted in decreased deposition of Aβ with a concomitant activation and accumulation of microglia around the senile plaques in the cortex and hippocampus (Wang et al. 2012). In an in vitro study, CXCL12 protected primary mouse neurons against Aβ-induced apoptosis and dendritic regression. This effect was mediated by activation of Akt and ERK1/2 kinases and stabilization of metalloproteinase ADAM17 activity. Similar changes, along with a significant decrease of oxidative damage, were demonstrated by in vivo studies on C57BL/6 mice treated intracerebroventricularly with CXCL12 and then with Aβ (Raman et al. 2011).

The involvement of the CXCL12/CXCR4 axis in microglial migration toward Aβ was confirmed in in vitro studies on neuronal and glial cells differentiated from neuronal stem cells and cultured in the central chamber of microfluidic device. To develop early AD and late AD models, the cells were incubated in the device for 0.5 weeks (3-week AD model) and 7.5 weeks (9-week AD model). To test the activation and migration of microglia cells by Aβ, they were loaded in the angular chamber and the number of recruited microglia in the central chamber was monitored. Migration of microglia toward neuronal and astrocytes cell cultures was blocked by AMD3100, a selective CXCR4 antagonist, indicating an involvement of CXCR4 in this process (McQuade et al. 2020).

In 5xFAD mice, the intraparenchymal delivery of human painless nerve growth factor (hNGFp) did not decrease the amyloid-β plaque load, but the same dose of the factor delivered intranasally, which was then widely biodistributed in the brain, showed a potent anti-amyloidogenic action and rescued synaptic plasticity and memory deficits. In addition, hNGFp given intranasally significantly decreased the expression of presenilin 1, nicastrin, and β-secretase-1 (BACE1); increased levels of full-length APP; and reduced amounts of C99 and C83/C89 terminal fragments of APP. It was demonstrated that hNGF acts on microglia and, to a lesser extent, on astrocytes. The neuroprotective effects of hNGFp were mediated by CXCL12, as the reduction of memory deficits, Aβ plaque load and Aβ oligomers level were blocked by AMD3100. AMD3100 also blocked the hNGFp-induced modulation of presenilin 1, BACE1, and APP processing products. Accordingly, CXCL12 decreased amyloid-β oligomer immunoreactivity in 5xFAD cultured cortical neurons and protected the wide-type neurons against Aβ-induced cytotoxicity (Capsoni et al. 2017).

CXCL12 was also involved in beneficial effects of mobilization of bone marrow mesenchymal stem cells following granulocyte colony-stimulating factor (G-CSF) treatment in APP_Swe_ or wide-type mice treated with Aβ. It has been documented that CXCL12/CXCR4-mediated chemotaxis leads to the recruitment and infiltration of repair-competent cells, resulting in enhanced neurogenesis and cognitive improvement (Wu et al. 2017).

#### CXCR3: CXCL9, CXCL10 (IP-10), CXCL11

CXCL10 (also known as Interferon gamma-induced protein 10 (IP-10) or small-inducible cytokine B10) is expressed on astrocytes in normal and AD brains, where it plays a role in their migration, while its receptor, CXCR3, is constitutively expressed on neurons. In AD brains, the expression of CXCL10 on astrocytes was upregulated as compared to normal brains. CXCL10-expressing astrocytes were accumulated around senile plaques and characterized by increased expression of another chemokine, CCL4. Similarly, hippocampal astrocytes cultured in vitro produce increased amounts of CXCL10 in response to Aβ stimulation (Lai et al. 2013; Xia et al. 2000). A meta-analysis revealed that plasma concentration of CXCL10 was significantly elevated in AD patients as compared to healthy controls (Lai et al. 2017).

Concentration of CXCL10 in CSF was higher in patients with mild AD, but not in those presenting severe AD (MMSE < 15), as compared to age-matched control subjects. In AD patients, levels of CXCL10 were positively correlated with MMSE score, suggesting that although they increased at the early stage of disease, but dropped drop as the impairment progresses. In this study, the control group included patients without memory complaints; however, with other noninflammatory neurological diseases, an association between elevated levels of CXCL10 and other pathologies cannot be excluded (Galimberti et al. 2006). In another study, CSF concentrations of CXCL10 did not differ between AD patients and the control group (patients submitted to elective surgery, without dementia, infections, autoimmune diseases, or cancer); however, CXCL10 levels in the AD group correlated positively with CSF levels of Aβ. Nevertheless, in this study, no stratification for the severity of AD was done, thus it cannot be concluded whether CXCL10 levels were different at the early stage of the disease (Corrêa et al. 2011).

By analogy to humans, elevated levels CXCL10 were found in the cerebral cortex and hippocampus of Tg2576 mice harboring human APP_Swe_ mutation, with intense expression of CXCL10 co-localizing with Aβ plaques (Duan et al. 2008). Increased expression of CXCL10, CXCR3 receptor, and its other ligand CXCL9 was also observed in 3xTg and APP/PS1 mice (Krauthausen et al. 2015; Martin et al. 2019; Zaheer et al. 2013). In female 5xFAD mice, CXCL10 was the earliest pro-inflammatory molecule induced as early as at 3 months of age, which is approx. 1 month after these mice begin to develop Aβ deposits. High levels of Aβ1-40 and Aβ1-42 were found in in the brain and CSF of 5xFAD mice; they even increase over age. Histological analyses of the cortex and hippocampus revealed a dramatic plaque load and β-sheet formation accompanied by strong neuroinflammation. The expression of CXCL10 in these Tg mice lines was found to further increase with age, which is paralleled with growing Aβ deposition, augmenting upregulation of inflammatory cytokines (IL-1β, TNF-α), astro- and microgliosis (Manji et al. 2019). The increased levels of CXCL10 in different Tg murine models of Aβ pathology is likely mediated by stimulation of microglia, as an in vitro study demonstrated that fibrillar Aβ stimulated production of CXCL10 by primary mouse microglia (Krauthausen et al. 2015).

In APP/PS1 mice, deficiency of CXCR3 resulted in reduction of Aβ and alleviation of plaque burden, concomitant with reduced activation and accumulation of astrocytes and microglia around plaques, and decreased levels of pro-inflammatory cytokines but increased expression of BDNF. These effects were paralleled with cognitive improvement observed in CXCR3^−/−^ APP/PS1 mice as compared to CXCR3^+/+^ APP/PS1 controls, suggesting beneficial effects of CXCL9, CXCL10, and BDNF alternations. The reduction of Aβ levels in CXCR3^−/−^ mice was found to be mediated by increased microglial phagocytosis, without significant changes of APP processing. The influence of CXCR3 on the microglial uptake of Aβ was also confirmed in vitro using a receptor 2-iminobenzimidazole antagonist, which increased the phagocytosis, as well as in a different in vivo model in which microglial phagocytosis of Aβ after intracerebral injection was enhanced in CXCR3^−/−^ mice (Krauthausen et al. 2015).

### CCL11: CCR3, CCR5 and CCR2

CCL11, also known as eotaxin-1, can act as either agonist of CCR3 and CCR5 or as an antagonist of CCR2. Its main effect is induction of eosinophil chemotaxis by interaction with CCR3. By blocking CCR2, this chemokine can inhibit CCL2-dependent chemotaxis of monocytes. Apart from CCL11, ligands of CCR3 include CCL5 (RANTES), CCL7 (MCP-3), and CCL13 (MCP-4) and CCL26 (eotaxin-3) (Ogilvie et al. 2001; Xia et al. 1998; Zhu et al. 2017). In the murine brain, CCL11 is mainly expressed in activated microglia (Zhu et al. 2017). Elevated expression of CCR3 and CCR5 was found in reactive microglia extracted from brains of AD patients (Xia et al. 1998). CCL11 levels were increased in plasma of AD patients (Choi et al. 2008) as compared to healthy, age-matched controls. Concentrations of CCL11 were also elevated in brains and CSF of APP/PS1 mice compared to wide-type controls. In both wide-type and APP/PS1 mice, CSF levels of CCL11 increased with age; however, this change was more pronounced in AD mice (Martin et al. 2019; Zhu et al.^2017^).

Recent studies suggest the negative impact of the CCL11/CCR3 axis on the AD pathology. In mouse hippocampal neuronal cultures, treatment with CLL11 induced production of Aβ1-42, tau hyperphosphorylation, and dendritic spine loss. Aggravation of Aβ and tau pathology by CCL11 was mediated, at least partially, by CCR3-dependent activation of two major kinases playing a key role in hyperphosphorylation of tau and APP processing, i.e., cyclin-dependent kinase 5 (CDK5) and glycogen synthase kinase-3β (GSK-3β) (Zhu et al. 2017). In APP/PS1 mice, knock-out of CCR3 reduced activity of GSK-3β and CDK5, leading to decreased tau hyperphosphorylation and Aβ deposition, along with reduced astro- and microgliosis, synaptic loss, and improvement in spatial learning and memory were found (Zhu et al. 2017). Treatment of APP/PS1 mice with YM344031, a BBB-penetrating CCR3 antagonist, resulted in marked attenuation of Aβ deposition and tau phosphorylation, processes dependent on the decreased APP processing and reduced activity of CDK5 and GSK-3β. YM344031 also prevented overt gliosis and synaptic loss, leading to reduction of cognitive impairment (Sui et al. 2019). The negative effect of CCL11 on cognition was also observed in wide-type mice. Treatment with a recombinant murine CCL11 resulted in impaired adult hippocampal neurogenesis leading to memory and learning deficits, while administration of CCL11 antibodies prevented such effects (Villeda et al. 2011). As CCR3 is present on microglia, it cannot be excluded that the neuroprotective effects of CCL11/CCR3 blockade result not only from reduced generation of Aβ and p-tau but also from decreased activation of microglia, sparing neurons, and synapses from cleavage (Sui et al. 2019; Zhu et al. 2017).

Changes in expression of chemokines and chemokine receptors in humans and mouse models of AD are summarized in Tables 1 and 2, respectively, while Table 3 presents a summary of effects of chemokine and chemokine receptor manipulations in rodent models of AD.

**Table 1:** Changes in expression of chemokines and chemokine receptors in humans

Chemokine/receptor	Patient group	Medium	Change in expression	References
CX3CL1	AD patients	Hippocampus and frontal cortex	Decrease	Cho et al. 2011
	MCI and AD	CSF	Decrease	Perea et al. 2018
	Severe AD (Braak V-VI)	Hippocampus	Decrease	Strobel et al. 2015
	Early AD (Braak II-IV)	Hippocampus	Increase	Lastres-Becker et al. 2014
	Severe AD (Braak V-VI)	Hippocampus	Increase	Dworzak et al. 2015
CCL2	AD patients	Plasma and CSF Brain tissue Hippocampus Frontal and temporal cortex	Increase	Corrêa et al. 2011 Galimberti et al. 2006 Lee et al. 2018 Liao et al. 2016 Nordengen et al. 2019 Sokolova et al. 2009 Stuart and Baune 2014
CCL3	AD patients	Hippocampus Temporal and frontal regions of brain	Increase	Liao et al. 2016
		Peripheral blood	Decrease	Geppert et al. 2010
CCL5	AD patients	Plasma Brain microvessels	Decrease	Tripathy et al. 2010 Vacinova et al. 2021
		Peripheral blood	Decrease (mRNA)	Kester et al. 2011
CCR5	AD patients	Reactive microglia Lymphocytes T and B	Increase	Bakshi et al. 2011 Pellicanò et al. 2010
CXCL1	AD patients	Peripheral monocytes	Increase	Zhang et al. 2013
CXCL8	AD patients	Brain tissue Neurons around Aβ plaques Plasma CSF	Increase	Alsadany et al. 2013 Ashutosh et al. 2011 Corrêa et al. 2011 Galimberti et al. 2006 Sokolova et al. 2009
CXCR2	AD patients	Brain Peripheral T lymphocytes Microglia around Aβ plaques	Increase	Liu et al. 2010a Ryu et al. 2015 Xia et al. 1997
CXCL12	Early-stage AD patients	Plasma	Decrease	Laske et al. 2008 Sanfilippo et al. 2020
	AD patients	Brain		
CXCR4	AD patients	Brain	Increase	Sanfilippo et al. 2020
CXCL10	AD patients	Brain Plasma	Increase	Lai et al. 2013 Lai et al. 2017 Xia et al. 2000
		CSF	Increase (mild AD) No change (severe AD)	Galimberti et al. 2006
			No change	Corrêa et al. 2011
CCR3	AD patients	Brain	Increase	Xia et al. 1998
CCL11	AD patients	Plasma	Increase	Choi et al. 2008

**Table 2:** Changes of expression of chemokines and chemokine receptors in mouse models of AD

Chemokine/receptor	Model	Changes in expression	Tissue	References
CX3CL1	Tg2576 mice at 9 and 17 months	↓ CX3CL1	Cerebral cortex	Duan et al. 2008
CCL2	5xFAD Tg2576 3xTg APP/PS1 mice	↑ CCL2	Brain	Manji et al. 2019 Hartlage-Rübsamen et al. 2015 Reale et al. 2018 Zaheer et al. 2013
CCR2	APP/PS1 mice	↑ CCR2	Brain	Krauthausen et al. 2015
CCL3 CCL4 CCL5	APP/PS1 mice	↑ CCL3 and CCL4	Brain	Jorda et al. 2019 Martin et al. 2019 Zhu et al. 2014
	THY-Tau22 mice	↑ CCL3, CCL4 and CCL5	Hippocampus	Laurent et al. 2017
	APP/PS1 mice	↑ CCL5	Brain	Martin et al. 2019
CCR5	APP/PS1 mice	↓ CCR5	Brain	Jorda et al. 2019
CXCL1 CXCL2	APP/PS1 mice	↑ CXCL1 and CXCL2	Brain	Martin et al. 2019 Watson and Fan 2005
CXCL12	Tg2576 mice	↓ CXCL12	Hippocampus	Parachikova and Cotman 2007
	Tg2576 mice	↑ CXCL12	brain	Wu et al. 2017
CXCR4	rTg4510 mice	↑ CXCR4	Brain regions affected by tau pathology	Bonham et al. 2018
	APP/PS1 mice	↑ CXCR4	Brain	Krauthausen et al. 2015
	Tg2576 mice	↓ CXCR4	Hippocampus	Parachikova and Cotman 2007
CXCL9 CXCL10	Tg2576 mice	↑ CXCL10	Cortex and hippocampus co-localization with Aβ plaques	Duan et al. 2008
	3xTg mice	↑ CXCL10	Brain	Zaheer et al. 2013
	5xFAD mice			Manji et al. 2019
	APP/PS1 mice			Martin et al. 2019
	APP/PS1 mice	↑ CXCL9, CXCL10	Brain (mRNA)	Krauthausen et al. 2015
CXCR3	APP/PS1 mice	↑ CXCR3	Brain (mRNA)	Krauthausen et al. 2015
CCL11	APP/PS1 mice	↑ CCL11	Brain and CSF	Martin et al. 2019 Zhu et al. 2017

↓ decrease, ↑ increase, *CSF* cerebrospinal fluid

**Table 3:** Effects of chemokine and chemokine receptor manipulations in rodent models of AD

Chemokine/receptor	Intervention	Model	Outcome	References
CX3CL1	Stereotactical injection of tau into the hippocampus	WT mice	Increased levels of CX3CL1	Lastres-Becker et al. 2014
	CX3CL1 knock-out	APP/PS1 mice	Increased tau phosphorylation Reduced Aβ accumulation	Lee et al. 2014
	Deletion of C-terminal membrane-bound fragment of CX3CL1	APP/PS1 mice	Increased neuroinflammation Increased tau phosphorylarion	Lee et al. 2014
	Deletion of C-terminal membrane-bound fragment of CX3CL1	hTau^+/−^; Mapt^−/−^ mice	Reduced CX3CR1 expression Increased neuroinflammation	Bemiller et al. 2018
	CX3CL1 overexpression	PS19 mice	Reduced neurodegeneration Cognitive improvement Extended lifespan	Fan et al. 2020
	Overexpression of C-terminal membrane-bound fragment of CX3CL1	5xFAD mice	Reduced Aβ deposition Decreased APP expression Decreased neuronal loss Increased neurogenesis	Fan et al. 2019
	Overexpression of soluble CX3CL1 fragment	rTG4510 mice (3–6 months)	Reduced tau pathology Reduced microglial activation Reduced neurodegeneration No cognitive improvement	Nash et al. 2013
	Overexpression of soluble CX3CL1 fragment	rTG4510 mice (7–8 months)	Cognitive improvement No effect on tau pathology No effect on neurodegeneration	Finneran et al. 2019
	Overexpression of soluble CX3CL1 fragment	APP/PS1 mice	No changes in Aβ pathology	Nash et al. 2013
CX3CR1	Injection of Aβ into the hippocampus	WT rats	Increased expression of CX3CR1	Wu et al. 2013
		WT rats treated with CX3CR1 siRNA	Reduced microglial activation, neuroinflammation and cognitive impairment	
	CX3CR1 knock-out	hAPP-J20 mice	Exacerbated tau pathology Worsened cognitive decline Increased IL-6	Cho et al. 2011
	CX3CR1 knock-out	hTau mice	Exacerbated tau pathology Worsened cognitive decline Increased microglial activation	Bhaksar et al. 2010
	CX3CR1 knock-out	CRND8 mice (APP_Swe_, APP_V717F_)	Reduced Aβ levels Increased microglial accumulation around Aβ plaques Increased Aβ phagocytosis by microglia	Liu et al. 2010b
	CX3CR1 knock-out	3xTg mice	Decreased neuronal loss No effect on Aβ accumulation and phagocytosis	Fuhrmann et al. 2010
	CX3CR1 deficiency (*CX3CR*^+*/*−^ *and CX3CR*^−*/*−^)	APP/PS1 mice APP_Swe_ mice	Reduced Aβ levels Reduced microglial activation Reduced microglial accumulation around Aβ plaques Increased Aβ phagocytosis by microglia	Lee et al. 2010
	CX3CR1 heterozygosity	APP/PS1 mice	Reduced Aβ levels Cognitive improvement	Hickman et al. 2019
CCL2	CCL2 knock-out	APP/PS1 mice	Increased levels of Aβ oligomers Impaired Aβ phagocytosis by microglia Increased cognitive decline Impaired neurogenesis	Kiyota et al. 2013
	CCL2 knock-out	PS1 mice	Impaired neurogenesis Impaired synaptic plasticity Increased cognitive impairment	Kiyota et al. 2015
	CCL2 suppression (AAV-mediated delivery of dominant-negative CCL2 mutant)	APP/PS1 mice	Decreased microglia activation Decreased levels of Aβ oligomers and fibrils Cognitive improvement	Kiyota et al. 2009a
	CCL2 knock-out	5xFAD mice	Reduced inflammation Reduced accumulation of Aβ plaques Reduced neuronal loss Cognitive improvement	Gutiérrez et al. 2019a, b
	CCL2 overexpression	APP mice	Increased Aβ deposition Increased accumulation of microglia around plaques Increased phagocytosis of Aβ Increased Aβ oligomerization in microglia Increased cognitive decline	Kiyota et al. 2009b Yamamoto et al. 2005
	CCL2 overexpression	rTg4510 mice	Increased microglia activation Aggravated tau pathology	Joly-Amado et al. 2020
CCR2	CCR2 knock-out	APP/PS1 mice	Increased oligomeric Aβ accumulation Increased cognitive decline Increased microglia accumulation around plaques Decreased microglia activation and phagocytosis	Naert and Rivest 2011
	CCR2 knock-out	Tg2576 mice	Decreased Aβ clearance Increased accumulation of Aβ	Mildner et al. 2011
	CCR2 knock-out	Tg2576 mice	Increased Aβ accumulation Decreased Aβ clearance by microglia Premature death	El Khoury et al. 2007
	Transplantation of bone marrow from CCR2 knock-out mice	APP/PS1 mice	Increased levels of Aβ oligomers Increased cognitive decline	Naert and Rivest 2012
CCL3 CCL4 CCL5	Hippocampal injection of Aβ	WT rats	Increased expression of CCL3 in peripheral T lymphocytes	Man et al. 2007
	ICV injection of Aβ(1–40)	WT mice	Increased expression of CCL3	Passos et al. 2009
	ICV injection of Aβ(1–40)	CCL3 knock-out mice	Protection from astrocytosis, microgliosis and neuroinflammation Reduced cognitive impairment	Passos et al. 2009
CCR5	Hippocampal injection of Aβ	WT rats	Increased CCR5 expression in brain endothelial cells	Li et al. 2009
	ICV injection of Aβ(1–40)	CCR5 knock-out mice	Protection from astrocytosis, microgliosis and neuroinflammation Reduced cognitive impairment	Passos et al. 2009
CXCL1 CXCL2	Injection of lentiviral CXCL1 into the hippocampus	Aged WT mice	Increased tau cleavage	Zhang et al. 2015
CXCL8	Injection of Aβ(1–42) into the hippocampus	WT rats	Increased expression of CXCL8 Gliosis Accumulation of T cells in brain Neuronal loss	Liu et al. 2010a Ryu et al. 2015
CXCR2	Injection of Aβ(1–42) into the hippocampus	WT rats	Increased expression of CXCR2 Gliosis Accumulation of T cells in brain Neuronal loss	Liu et al. 2010a Ryu et al. 2015
	Treatment with selective CXCR2 antagonist SB225002	APP_Swe_ mice	Reduced levels of soluble Aβ(1–40)	Bakshi et al. 2008, 2011
CXCL12	Injection of Aβ into the brain	WT mice	Increased levels of CXCL12 in injected regions	Wu et al. 2017
	ICV injection of CXCL12	APP/PS1 mice	Decreased accumulation of Aβ Increased accumulation and activation of microglia around Aβ plaques	Wang et al. 2012
	ICV pretreatment with CXCL12 ICV treatment with Aβ	WT mice	Decreased neuronal loss Decreased oxidative damage	Raman et al. 2011
CXCR4	Intranasal treatment with hNGFp Treatment with CXCR4 antagonist AMD3100	5xFAD mice	Blockade of CXCR4 resulted in abolishment of hNGFp-induced reduction of cognitive impairment, Aβ plaque load and levels of Aβ oligomers	Capsoni et al. 2017
CXCR3	CXCR3 knock-out	APP/PS1 mice	Reduced Aβ levels and pathology Reduced gliosis around Aβ plaques Increased microglial phagocytosis of Aβ Reduced neuroinflammation Increased expression of BDNF Cognitive improvement	Krauthausen et al. 2015
	CXCR3 knock-out Intracerebral Aβ injection	WT mice	Increased microglial phagocytosis of Aβ	
CCR3	CCR3 knock-out	APP/PS1 mice	Decreased tau phosphorylation Decreased Aβ accumulation Reduced gliosis Cognitive improvement	Zhu et al. 2017
	Treatment with selective CCR3 antagonist YM344031	APP/PS1 mice	Decreased tau phosphorylation Decreased Aβ accumulation Reduced gliosis Reduced synaptic loss Cognitive improvement	Sui et al. 2019

## Closing Thoughts

The linkage between pathologic alterations in AD brain and intensified neuroinflammatory processes mediated by chemokines is well established. Nevertheless, precise mechanisms need to be fully elucidated. Although in physiological condition, chemokines maintain homeostasis in the brain regulating cellular communication, neuronal activity, and survival, constant overproduction of chemokines leads to increased deposition of senile plaque and NFTs in the aftermath of sustained neuroinflammatory processes. On the other hand, chemokines also prevent the build-up of brain deposits by clearing pathological lesions. Therefore, deficiency or complete removal of chemokines results in exacerbation of AD. The extensive studies carried out on cell lines, transgenic animal models or with human participants provide new insights into the role distinct chemokines play in the development of AD. Controlled modulation of chemokines may provide plausible therapeutic strategies for preventing neuronal loss and slowing or even halting progression of AD.

## Data Availability

Not applicable.

## References

[CR1] Alsadany MA, Shehata HH, Mohamad MI, Mahfouz RG (2013) Histone Deacetylases Enzyme, Copper, and IL-8 Levels in Patients With Alzheimer’s Disease. Am J Alzheimer's Dis & Other Dementiasr. 28(1):54-61. 10.1177/1533317512467680 10.1177/1533317512467680 PMC10697231 23242124

[CR2] Armstrong R (2019). Risk factors for Alzheimer’s disease. Folia Neuropathol.

[CR3] Ashford JW, Salehi A, Furst A, Bayley P, Frisoni GB, Jack CR, Sabri O, Adamson MM, Coburn KL, Olichey J, Schuff N, Spielman D, Edland SD, Black S, Rosen A, Kennedy D, Weiner M, Perry G (2011). Imaging the Alzheimer brain. Journal of Alzheimer’s Disease.

[CR4] Ashutosh KW, Cotter R, Borgmann K, Wu L, Persidsky R, Sakhuja N, Ghorpade A (2011). CXCL8 protects human neurons from amyloid-β-induced neurotoxicity: relevance to Alzheimer's disease. Biochem Biophys Res Commun.

[CR5] Atri A (2019). The Alzheimer’s disease clinical spectrum. Diagnosis and Management. Med Clin N Am.

[CR6] Ayodele T, Rogaeva E, Kurup JT, Beecham G, Reitz C (2021). Early-onset Alzheimer’s disease: what is missing in research?. Curr Neurol Neurosci Rep.

[CR7] Bachstetter AD, Morganti JM, Jernberg J, Schlunk A, Mitchell SH, Brewster KW, Hudson CE, Cole MJ, Harrison JK, Bickford PC, Gemma C (2011). Fractalkine and CX 3 CR1 regulate hippocampal neurogenesis in adult and aged rats. Neurobiol Aging.

[CR8] Bajetto A, Bonavia R, Barbero S, Schettini G (2002). Characterization of chemokines and their receptors in the central nervous system: physiopathological implications. J Neurochem.

[CR9] Bakshi P, Margenthaler E, Laporte V, Crawford F, Mullan M (2008). Novel role of CXCR2 in regulation of gamma-secretase activity. ACS Chem Biol.

[CR10] Bakshi P, Margenthaler E, Reed J, Crawford F, Mullan M (2011). Depletion of CXCR2 inhibits γ-secretase activity and amyloid-β production in a murine model of Alzheimer's disease. Cytokine.

[CR11] Balistreri CR, Grimaldi MP, Vasto S, Listi F, Chiappelli M, Licastro F, Lio D, Caruso C, Candore G (2006). Association between the polymorphism of CCR5 and Alzheimer's disease: results of a study performed on male and female patients from Northern Italy. Ann N Y Acad Sci.

[CR12] Bazan JF, Bacon KB, Hardiman G, Wang W, Soo K, Rossi D, Greaves DR, Zlotnik A, Schall TJ (1997). A new class of membrane-bound chemokine with a CX3C motif. Nature.

[CR13] Bemiller SM, Maphis NM, Formica SV, Wilson GN, Miller CM, Xu G, Kokiko-Cochran ON, Kim KW, Jung S, Cannon JL, Crish SD, Cardona AE, Lamb BT, Bhaskar K (2018). Genetically enhancing the expression of chemokine domain of CX3CL1 fails to prevent tau pathology in mouse models of tauopathy. J Neuroinflammation.

[CR14] Bhaskar K, Konerth M, Kokiko-Cochran ON, Cardona A, Ransohoff RM, Lamb BT (2010). Regulation of tau pathology by the microglial fractalkine receptor. Neuron.

[CR15] Bisht K, Sharma KP, Lecours C, Sánchez MG, El Hajj H, Milior G, Olmos-Alonso A, Gómez-Nicola D, Luheshi G, Vallières L, Branchi I, Maggi L, Limatola C, Butovsky O, Tremblay MÈ (2016). Dark microglia: a new phenotype predominantly associated with pathological states. Glia.

[CR16] Bolós M, Llorens-Martín M, Perea JR, Jurado-Arjona J, Rábano A, Hernández F, Avila J (2017). Absence of CX3CR1 impairs the internalization of Tau by microglia. Mol Neurodegener.

[CR17] Bonham LW, Karch CM, Fan CC, Tan C, Geier EG, Wang Y, Wen N, Broce IJ, Li Y, Barkovich MJ, Ferrari R, Hardy J, Momeni P, Höglinger G, Müller U, Hess CP, Sugrue LP, Dillon WP, Schellenberg GD, Miller BL, Andreassen OA, Dale AM, Barkovich AJ, Yokoyama JS, Desikan RS; International FTD-Genomics Consortium (IFGC); International Parkinson’s Disease Genetics Consortium (IPDGC); International Genomics of Alzheimer’s Project (IGAP) (2018). CXCR4 involvement in neurodegenerative diseases. Transl Psychiatry.

[CR18] Brunello CA, Merezhko M, Uronen R-L, Huttunen HJ (2019). Mechanisms of secretion and spreading of pathological tau protein. Cell Mol Life Sci.

[CR19] Brunholz S, Sisodia S, Lorenzo A, Deyts C, Kins S, Morfini G (2012). Axonal transport of APP and the spatial regulation of APP cleavage and function in neuronal cells. Exp Brain Res.

[CR20] Capsoni S, Malerba F, Carucci NM, Rizzi C, Criscuolo C, Origlia N, Calvello M, Viegi A, Meli G, Cattaneo A (2017). The chemokine CXCL12 mediates the anti-amyloidogenic action of painless human nerve growth factor. Brain.

[CR21] Chidambaram H, Das R, Chinnathambi S (2020). Interaction of Tau with the chemokine receptor, CX3CR1 and its effect on microglial activation, migration and proliferation. Cell Biosci.

[CR22] Cho SH, Sun B, Zhou Y, Kauppinen TM, Halabisky B, Wes P, Ransohoff RM, Gan L (2011). CX3CR1 protein signaling modulates microglial activation and protects against plaque-independent cognitive deficits in a mouse model of Alzheimer disease. J Biol Chem.

[CR23] Choi C, Jeong JH, Jang JS, Choi K, Lee J, Kwon J, Choi KG, Lee JS, Kang SW (2008). Multiplex analysis of cytokines in the serum and cerebrospinal fluid of patients with Alzheimer's disease by color-coded bead technology. J Clin Neurol.

[CR24] Chu HX, Arumugam TV, Gelderblom M, Magnus T, Drummond GR, Sobey CG (2014). Role of CCR2 in inflammatory conditions of the central nervous system. J Cereb Blood Flow Metab.

[CR25] Combarros O, Infante J, Llorca J, Peña N, Fernández-Viadero C, Berciano J (2004). The chemokine receptor CCR5-Delta32 gene mutation is not protective against Alzheimer's disease. Neurosci Lett.

[CR26] Coronel R, Bernabeu-Zornoza A, Palmer C, Muñiz-Moreno M, Zambrano A, Cano E, Liste I (2018). Role of amyloid precursor protein (APP) and its derivatives in the biology and cell fate specification of neural stem cells. Mol Neurobiol.

[CR27] Corrêa JD, Starling D, Teixeira AL, Caramelli P, Silva TA (2011). Chemokines in CSF of Alzheimer's disease patients. Arq Neuropsiquiatr.

[CR28] Cudaback E, Yang Y, Montine TJ, Keene CD (2015). APOE genotype-dependent modulation of astrocyte chemokine CCL3 production. Glia.

[CR29] Duan RS, Yang X, Chen ZG, Lu MO, Morris C, Winblad B, Zhu J (2008). Decreased fractalkine and increased IP-10 expression in aged brain of APP(swe) transgenic mice. Neurochem Res.

[CR30] Dworzak J, Renvoisé B, Habchi J, Yates EV, Combadière C, Knowles TP, Dobson CM, Blackstone C, Paulsen O, Murphy PM (2015). Neuronal Cx3cr1 deficiency protects against amyloid β-induced neurotoxicity. PLoS ONE.

[CR31] El Khoury JB, Moore KJ, Means TK, Leung J, Terada K, Toft M, Freeman MW, Luster AD (2003). CD36 mediates the innate host response to beta-amyloid. J Exp Med.

[CR32] El Khoury J, Toft M, Hickman SE, Means TK, Terada K, Geula C, Luster AD (2007). Ccr2 deficiency impairs microglial accumulation and accelerates progression of Alzheimer-like disease. Nat Med.

[CR33] Fan Q, Gayen M, Singh N, Gao F, He W, Hu X, Tsai LH, Yan R (2019). The intracellular domain of CX3CL1 regulates adult neurogenesis and Alzheimer's amyloid pathology. J Exp Med.

[CR34] Fan Q, He W, Gayen M, Benoit MR, Luo X, Hu X, Yan R (2020). Activated CX3CL1/Smad2 signals prevent neuronal loss and Alzheimer's tau pathology-mediated cognitive dysfunction. J Neurosci.

[CR35] Finneran DJ, Morgan D, Gordon MN, Nash KR (2019). CNS-wide over expression of fractalkine improves cognitive functioning in a tauopathy model. J Neuroimmune Pharmacol.

[CR36] Fuhrmann M, Bittner T, Jung CK, Burgold S, Page RM, Mitteregger G, Haass C, LaFerla FM, Kretzschmar H, Herms J (2010) Microglial Cx3cr1 knockout prevents neuron loss in a mouse model of Alzheimer's disease. Nat Neurosci 13 4 411 413. 10.1038/nn.2511 PMC4072212 20305648

[CR37] Galimberti D, Schoonenboom N, Scheltens P, Fenoglio C, Bouwman F, Venturelli E, Guidi I, Blankenstein MA, Bresolin N, Scarpini E (2006). Intrathecal chemokine synthesis in mild cognitive impairment and Alzheimer disease. Arch Neurol.

[CR38] Geppert AM, Losy J, Przedpelska-ObWuer E, Kozubski W (2010). CCL3 correlates with the number of mood disturbances and personality changes in patients with Alzheimer's disease. Psychiatry Res.

[CR39] Giri RK, Rajagopal V, Shahi S, Zlokovic BV, Kalra VK (2005). Mechanism of amyloid peptide induced CCR5 expression in monocytes and its inhibition by siRNA for Egr-1. Am J Physiol Cell Physiol.

[CR40] Gutiérrez IL, González-Prieto M, Caso JR, García-Bueno B, Leza JC, Madrigal JLM (2019). Reboxetine treatment reduces neuroinflammation and neurodegeneration in the 5xFAD mouse model of Alzheimer's disease: role of CCL2. Mol Neurobiol.

[CR41] Hall AM, Roberson ED (2012). Mouse models of Alzheimer's disease. Brain Res Bull.

[CR42] Hampel H, Mesulam MM, Cuello AC, Farlow MR, Giacobini E, Grossberg GT, Khachaturian AS, Vergallo A, Cavedo E, Snyder PJ, Khachaturian ZS (2018). The cholinergic system in the pathophysiology and treatment of Alzheimer’s disease. Brain.

[CR43] Hansen RA, Gartlehner G, Webb AP, Morgan LC, Moore CG, Jonas DE (2008). Efficacy and safety of donepezil, galantamine, and rivastigmine for the treatment of Alzheimer’s disease: a systematic review and meta-analysis. Clin Interv Aging.

[CR44] Hartlage-Rübsamen M, Waniek A, Meissner J, Morawski M, Schilling S, Jäger C, Kleinschmidt M, Cynis H, Kehlen A, Arendt T, Demuth HU, Rossner S (2015). Isoglutaminyl cyclase contributes to CCL2-driven neuroinflammation in Alzheimer's disease. Acta Neuropathol.

[CR45] Hickman SE, Allison EK, Coleman U, Kingery-Gallagher ND, El Khoury J (2019) Heterozygous CX3CR1 Deficiency in Microglia Restores Neuronal β-Amyloid Clearance Pathways and Slows Progression of Alzheimer's Like-Disease in PS1-APP Mice. Front Immunol 10:2780. 10.3389/fimmu.2019.02780 10.3389/fimmu.2019.02780 PMC6900980 31849963

[CR46] Gutiérrez SE, Allison EK, Coleman U, Kingery-Gallagher ND, El Khoury J (2019). Heterozygous CX3CR1 deficiency in microglia restores neuronal β-amyloid clearance pathways and slows progression of Alzheimer's like-disease in PS1-APP mice. Front Immunol.

[CR47] Huerta C, Alvarez V, Mata IF, Coto E, Ribacoba R, Martínez C, Blázquez M, Guisasola LM, Salvador C, Lahoz CH, Peña J (2004) Chemokines (RANTES and MCP-1) and chemokine-receptors (CCR2 and CCR5) gene polymorphisms in Alzheimer's and Parkinson's disease. Neurosci Lett 370(2–3):151–154. 10.1016/j.neulet.2004.08.016 10.1016/j.neulet.2004.08.016 15488313

[CR48] Hughes CE, Nibbs RJB (2018). A guide to chemokines and their receptors. FEBS J.

[CR49] Hwang CJ, Park MH, Hwang JY, Kim JH, Yun NY, Oh SY, Song JK, Seo HO, Kim YB, Hwang DY, Oh KW, Han SB, Hong JT (2016) CCR5 deficiency accelerates lipopolysaccharide-induced astrogliosis, amyloid-beta deposit and impaired memory function. Oncotarget 7(11):11984–11999. 10.18632/oncotarget.7453 10.18632/oncotarget.7453 PMC4914263 26910914

[CR50] Ishizuka K, Kimura T, Igata-yi R, Katsuragi S, Takamatsu J, Miyakawa T (1997). Identification of monocyte chemoattractant protein-1 in senile plaques and reactive microglia of Alzheimer's disease. Psychiatry Clin Neurosci.

[CR51] Ito S, Sawada M, Haneda M, Ishida Y, Isobe K (2006). Amyloid-beta peptides induce several chemokine mRNA expressions in the primary microglia and Ra2 cell line via the PI3K/Akt and/or ERK pathway. Neurosci Res.

[CR52] Joly-Amado A, Hunter J, Quadri Z, Zamudio F, Rocha-Rangel PV, Chan D, Kesarwani A, Nash K, Lee DC, Morgan D, Gordon MN, Selenica MB (2020). CCL2 Overexpression in the brain promotes glial activation and accelerates tau pathology in a mouse model of tauopathy. Front Immunol.

[CR53] Jorda A, Cauli O, Santonja JM, Aldasoro M, Aldasoro C, Obrador E, Vila JM, Mauricio MD, Iradi A, Guerra-Ojeda S, Marchio P, Valles SL (2019). Changes in chemokines and chemokine receptors expression in a mouse model of Alzheimer's disease. Int J Biol Sci.

[CR54] Jouanne M, Rault S, Voisin-Chiret A-S (2017). Tau protein aggregation in Alzheimer's disease: An attractive target for the development of novel therapeutic agents. Eur J Med Chem.

[CR55] Kester MI, van der Flier WM, Visser A, Blankenstein MA, Scheltens P, Oudejans CB (2011). Decreased mRNA expression of CCL5 [RANTES] in Alzheimer's disease blood samples. Clin Chem Lab Med.

[CR56] Kiyota T, Gendelman HE, Weir RA, Higgins EE, Zhang G, Jain M (2013). CCL2 affects β-amyloidosis and progressive neurocognitive dysfunction in a mouse model of Alzheimer's disease. Neurobiol Aging.

[CR57] Kiyota T, Morrison CM, Tu G, Dyavarshetty B, Weir RA, Zhang G, Xiong H, Gendelman HE (2015). Presenilin-1 familial Alzheimer's disease mutation alters hippocampal neurogenesis and memory function in CCL2 null mice. Brain Behav Immun.

[CR58] Kiyota T, Yamamoto M, Schroder B, Jacobsen MT, Swan RJ, Lambert MP, Klein WL, Gendelman HE, Ransohoff RM, Ikezu T (2009). AAV1/2-mediated CNS gene delivery of dominant-negative CCL2 mutant suppresses gliosis, beta-amyloidosis, and learning impairment of APP/PS1 mice. Mol Ther.

[CR59] Kiyota T, Yamamoto M, Xiong H, Lambert MP, Klein WL, Gendelman HE, Ransohoff RM, Ikezu T (2009). CCL2 accelerates microglia-mediated Abeta oligomer formation and progression of neurocognitive dysfunction. PLoS ONE.

[CR60] Krauthausen M, Kummer MP, Zimmermann J, Reyes-Irisarri E, Terwel D, Bulic B, Heneka MT, Müller M (2015). CXCR3 promotes plaque formation and behavioral deficits in an Alzheimer's disease model. J Clin Invest.

[CR61] Lai KSP, Liu CS, Rau A, Lanctôt KL, Köhler CA, Pakosh M, Carvalho AF, Herrmann N (2017). Peripheral inflammatory markers in Alzheimer's disease: a systematic review and meta-analysis of 175 studies. J Neurol Neurosurg Psychiatry.

[CR62] Lai W, Wu J, Zou X, Xie J, Zhang L, Zhao X, Zhao M, Wang Q, Ji J (2013). Secretome analyses of Aβ(1–42) stimulated hippocampal astrocytes reveal that CXCL10 is involved in astrocyte migration. J Proteome Res.

[CR63] Laske C, Stellos K, Eschweiler GW, Leyhe T, Gawaz M (2008). Decreased CXCL12 (SDF-1) plasma levels in early Alzheimer's disease: a contribution to a deficient hematopoietic brain support?. J Alzheimers Dis.

[CR64] Lastres-Becker I, Innamorato NG, Jaworski T, Rábano A, Kügler S, Van Leuven F, Cuadrado A (2014). Fractalkine activates NRF2/NFE2L2 and heme oxygenase 1 to restrain tauopathy-induced microgliosis. Brain.

[CR65] Laurent C, Dorothée G, Hunot S, Martin E, Monnet Y, Duchamp M, Dong Y, Légeron FP, Leboucher A, Burnouf S, Faivre E, Carvalho K, Caillierez R, Zommer N, Demeyer D, Jouy N, Sazdovitch V, Schraen-Maschke S, Delarasse C, Buée L, Blum D (2017). Hippocampal T cell infiltration promotes neuroinflammation and cognitive decline in a mouse model of tauopathy. Brain.

[CR66] Lee JK, Schuchman EH, Jin HK, Bae JS (2012). Soluble CCL5 derived from bone marrow-derived mesenchymal stem cells and activated by amyloid β ameliorates Alzheimer's disease in mice by recruiting bone marrow-induced microglia immune responses. Stem Cells.

[CR67] Lee S, Varvel NH, Konerth ME, Xu G, Cardona AE, Ransohoff RM, Lamb BT (2010). CX3CR1 deficiency alters microglial activation and reduces beta-amyloid deposition in two Alzheimer's disease mouse models. Am J Pathol.

[CR68] Lee S, Xu G, Jay TR, Bhatta S, Kim KW, Jung S, Landreth GE, Ransohoff RM, Lamb BT (2014). Opposing effects of membrane-anchored CX3CL1 on amyloid and tau pathologies via the p38 MAPK pathway. J Neurosci.

[CR69] Lee WJ, Liao YC, Wang YF, Lin IF, Wang SJ, Fuh JL (2018). Plasma MCP-1 and cognitive decline in patients with Alzheimer's disease and mild cognitive impairment: a two-year follow-up study. Sci Rep.

[CR70] Lee YK, Kwak DH, Oh KW, Nam SY, Lee BJ, Yun YW, Kim YB, Han SB, Hong JT (2009). CCR5 deficiency induces astrocyte activation, Aβ deposit and impaired memory function. Neurobiol Learn Mem.

[CR71] Li M, Shang DS, Zhao WD, Tian L, Li B, Fang WG, Zhu L, Man SM, Chen YH (2009). Amyloid β interaction with receptor for advanced glycation end products up-regulates brain endothelial CCR5 expression and promotes T cells crossing the blood-brain barrier. J Immunol.

[CR72] Liao Y, Qi XL, Cao Y, Yu WF, Ravid R, Winblad B, Pei JJ, Guan ZZ (2016). Elevations in the levels of NF-κB and inflammatory chemotactic factors in the brains with Alzheimer's disease— one mechanism may involve α3 nicotinic acetylcholine receptor. Curr Alzheimer Res.

[CR73] Liu YJ, Guo DW, Tian L, Shang DS, Zhao WD, Li B, Fang WG, Zhu L, Chen YH (2010). Peripheral T cells derived from Alzheimer's disease patients overexpress CXCR2 contributing to its transendothelial migration, which is microglial TNF-alpha-dependent. Neurobiol Aging.

[CR74] Liu Z, Condello C, Schain A, Harb R, Grutzendler J (2010). CX3CR1 in microglia regulates brain amyloid deposition through selective protofibrillar amyloid-β phagocytosis. J Neurosci.

[CR75] Man SM, Ma YR, Shang DS, Zhao WD, Li B, Guo DW, Fang WG, Zhu L, Chen YH (2007). Peripheral T cells overexpress MIP-1α to enhance its transendothelial migration in Alzheimer's disease. Neurobiol Aging.

[CR76] Manji Z, Rojas A, Wang W, Dingledine R, Varvel NH, Ganesh T (2019). 5xFAD mice display sex-dependent inflammatory gene induction during the prodromal stage of Alzheimer's disease. J Alzheimers Dis.

[CR77] Marciniak E, Faivre E, Dutar P, Alves Pires C, Demeyer D, Caillierez R, Laloux C, Buée L, Blum D, Humez S (2015). The Chemokine MIP-1α/CCL3 impairs mouse hippocampal synaptic transmission, plasticity and memory. Sci Rep.

[CR78] Martin E, Amar M, Dalle C, Youssef I, Boucher C, Le Duigou C, Brückner M, Prigent A, Sazdovitch V, Halle A, Kanellopoulos JM, Fontaine B, Delatour B, Delarasse C (2019). New role of P2X7 receptor in an Alzheimer's disease mouse model. Mol Psychiatry.

[CR79] Martin E, Boucher C, Fontaine B, Delarasse C (2017). Distinct inflammatory phenotypes of microglia and monocyte-derived macrophages in Alzheimer's disease models: effects of aging and amyloid pathology. Aging Cell.

[CR80] Matsunaga S, Kishi T, Nomura I, Sakuma K, Okuya M, Ikuta T, Iwata N (2018). The efficacy and safety of memantine for the treatment of Alzheimer's disease. Expert Opin Drug Saf.

[CR81] McKhann GM, Knopman DS, Chertkow H, Hyman BT, Jack CR, Kawas CH, Klunk WE, Koroshetz WJ, Manly JJ, Mayeux R, Mohs RC, Morris JC, Rossor MN, Scheltens P, Carillo MC, Thies B, Weintraub S, Phelps CH (2011). The diagnosis of dementia due to Alzheimer’s disease: recommendations from the National Institute on Aging-Alzheimer’s Association workgroups on diagnostic guidelines for Alzheimer’s disease. Alzheimers Dement.

[CR82] McQuade A, Kang YJ, Hasselmann J, Jairaman A, Sotelo A, Coburn M, Shabestari SK, Chadarevian JP, Fote G, Tu CH, Danhash E, Silva J, Martinez E, Cotman C, Prieto GA, Thompson LM, Steffan JS, Smith I, Davtyan H, Cahalan M, Cho H, Blurton-Jones M (2020). Gene expression and functional deficits underlie TREM2-knockout microglia responses in human models of Alzheimer's disease. Nat Commun.

[CR83] Mildner A, Schlevogt B, Kierdorf K, Böttcher C, Erny D, Kummer MP, Quinn M, Brück W, Bechmann I, Heneka MT, Priller J, Prinz M (2011). Distinct and non-redundant roles of microglia and myeloid subsets in mouse models of Alzheimer's disease. J Neurosci.

[CR84] Minter MR, Taylor JM, Crack PJ (2016). The contribution of neuroinflammation to amyloid toxicity in Alzheimer’s disease. J Neurochem.

[CR85] Murcia JDG, Weinert A, Freitas CMT, Arens DK, Ferrel MN, Grose JH, Ridge PG, Wilson E, Kauwe JSK, Weber KS (2020). Atypical chemokine receptor ACKR2-V41A has decreased CCL2 binding, scavenging, and activation, supporting sustained inflammation and increased Alzheimer's disease risk. Sci Rep.

[CR86] Naert G, Rivest S (2011). CC chemokine receptor 2 deficiency aggravates cognitive impairments and amyloid pathology in a transgenic mouse model of Alzheimer's disease. J Neurosci.

[CR87] Naert G, Rivest S (2012). Hematopoietic CC-chemokine receptor 2 (CCR2) competent cells are protective for the cognitive impairments and amyloid pathology in a transgenic mouse model of Alzheimer's disease. Mol Med.

[CR88] Nash KR, Lee DC, Hunt JB, Morganti JM, Selenica ML, Moran P, Reid P, Brownlow M, Guang-Yu Yang C, Savalia M, Gemma C, Bickford PC, Gordon MN, Morgan D (2013). Fractalkine overexpression suppresses tau pathology in a mouse model of tauopathy. Neurobiol Aging.

[CR89] Necula D, Riviere-Cazaux C, Shen Y, Zhou M (2021). Insight into the roles of CCR5 in learning and memory in normal and disordered states. Brain Behav Immun.

[CR90] Nordengen K, Kirsebom BE, Henjum K, Selnes P, Gísladóttir B, Wettergreen M, Torsetnes SB, Grøntvedt GR, Waterloo KK, Aarsland D, Nilsson LNG, Fladby T (2019). Glial activation and inflammation along the Alzheimer's disease continuum. J Neuroinflammation.

[CR91] Ogilvie P, Bardi G, Clark-Lewis I, Baggiolini M, Uguccioni M (2001). Eotaxin is a natural antagonist for CCR2 and an agonist for CCR5. Blood.

[CR92] Pan Y, Lloyd C, Zhou H, Dolich S, Deeds J, Gonzalo JA, Vath J, Gosselin M, Ma J, Dussault B, Woolf E, Alperin G, Culpepper J, Gutierrez-Ramos JC, Gearing D (1997). Neurotactin, a membrane-anchored chemokine upregulated in brain inflammation. Nature.

[CR93] Parachikova A, Cotman CW (2007). Reduced CXCL12/CXCR4 results in impaired learning and is downregulated in a mouse model of Alzheimer disease. Neurobiol Dis.

[CR94] Passos GF, Figueiredo CP, Prediger RD, Pandolfo P, Duarte FS, Medeiros R, Calixto JB (2009). Role of the macrophage inflammatory protein-1α/CC chemokine receptor 5 signaling pathway in the neuroinflammatory response and cognitive deficits induced by beta-amyloid peptide. Am J Pathol.

[CR95] Pellicanò M, Bulati M, Buffa S, Barbagallo M, Di Prima A, Misiano G, Picone P, Di Carlo M, Nuzzo D, Candore G, Vasto S, Lio D, Caruso C, Colonna-Romano G (2010). Systemic immune responses in Alzheimer's disease: in vitro mononuclear cell activation and cytokine production. J Alzheimers Dis.

[CR96] Perea JR, Lleó A, Alcolea D, Fortea J, Ávila J, Bolós M (2018). Decreased CX3CL1 levels in the cerebrospinal fluid of patients with Alzheimer's disease. Front Neurosci.

[CR97] Puzzo D, Gulisano W, Arancio O, Palmeri A (2015). The keystone of Alzheimer pathogenesis might be sought in Aβ physiology. Neuroscience.

[CR98] Qin B, Li L, Wang S, Wu J, Huang Y, Zhou P, Bai J, Zheng Y (2016). Interleukin-8 gene polymorphism -251T>A contributes to Alzheimer's disease susceptibility. Medicine (baltimore).

[CR99] Raman D, Milatovic SZ, Milatovic D, Splittgerber R, Fan GH, Richmond A (2011). Chemokines, macrophage inflammatory protein-2 and stromal cell-derived factor-1α, suppress amyloid β-induced neurotoxicity. Toxicol Appl Pharmacol.

[CR100] Rao YL, Ganaraja B, Murlimanju BV, Joy T, Krishnamurthy A, Agrawal A (2022) Hippocampus and its involvement in Alzheimer's disease: a review. 3 Biotech 12(2):55. 10.1007/s13205-022-03123-4 10.1007/s13205-022-03123-4 PMC8807768 35116217

[CR101] Reale M, D'Angelo C, Costantini E, Di Nicola M, Yarla NS, Kamal MA, Salvador N, Perry G (2018) Expression profiling of cytokine, cholinergic markers, and amyloid-β deposition in the APPSWE/PS1dE9 mouse model of Alzheimer's disease pathology. J Alzheimers Dis 62(1):467–476. 10.3233/JAD-170999 PMC5817902 29439355

[CR102] Rezazadeh M, Khorrami A, Yeghaneh T, Talebi M, Kiani SJ, Heshmati Y, Gharesouran J (2016). Genetic factors affecting late-onset Alzheimer's disease susceptibility. Neuromolecular Med.

[CR103] Roberts TK, Eugenin EA, Lopez L, Romero IA, Weksler BB, Couraud PO, Berman JW (2012). CCL2 disrupts the adherens junction: implications for neuroinflammation. Lab Invest.

[CR104] Rogers JT, Morganti JM, Bachstetter AD, Hudson CE, Peters MM, Grimmig BA, Weeber EJ, Bickford PC, Gemma C (2011). CX3CR1 deficiency leads to impairment of hippocampal cognitive function and synaptic plasticity. J Neurosci.

[CR105] Rosi S, Pert CB, Ruff MR, McGann-Gramling K, Wenk GL (2005). Chemokine receptor 5 antagonist D-Ala-peptide T-amide reduces microglia and astrocyte activation within the hippocampus in a neuroinflammatory rat model of Alzheimer's disease. Neuroscience.

[CR106] Ryu JK, Cho T, Choi HB, Jantaratnotai N, McLarnon JG (2015). Pharmacological antagonism of interleukin-8 receptor CXCR2 inhibits inflammatory reactivity and is neuroprotective in an animal model of Alzheimer's disease. J Neuroinflammation.

[CR107] Saido TC (2013) Metabolism of amyloid β peptide and pathogenesis of Alzheimer’s disease. Proc Jpn Acad Ser B Phys Biol Sci 89(7) 321:339. 10.2183/pjab.89.321 10.2183/pjab.89.321 PMC3758963 23883611

[CR108] Sanfilippo C, Castrogiovanni P, Imbesi R, Nunnari G, Di Rosa M (2020). Postsynaptic damage and microglial activation in AD patients could be linked CXCR4/CXCL12 expression levels. Brain Res.

[CR109] Sengoku R (2020). Aging and Alzheimer disease pathology. Neuropathology.

[CR110] Shang Y, Tian L, Chen T, Liu X, Zhang J, Liu D, Wei J, Fang W, Chen Y, Shang D (2019). CXCL1 promotes the proliferation of neural stem cells by stimulating the generation of reactive oxygen species in APP/PS1 mice. Biochem Biophys Res Commun.

[CR111] Skuljec J, Sun H, Pul R, Bénardais K, Ragancokova D, Moharregh-Khiabani D, Kotsiari A, Trebst C, Stangel M (2011). CCL5 induces a pro-inflammatory profile in microglia in vitro. Cell Immunol.

[CR112] Smits HA, Rijsmus A, van Loon JH, Wat JW, Verhoef J, Boven LA, Nottet HS (2002). Amyloid-β-induced chemokine production in primary human macrophages and astrocytes. J Neuroimmunol.

[CR113] Sokolova A, Hill MD, Rahimi F, Warden LA, Halliday GM, Shepherd CE (2009). Monocyte chemoattractant protein-1 plays a dominant role in the chronic inflammation observed in Alzheimer's disease. Brain Pathol.

[CR114] Streit WJ, Braak H, Del Tredici K, Leyh J, Lier J, Khoshbouei H, Eisenlӧffel C, Müller W, Bechmann I (2018). Microglial activation occurs late during preclinical Alzheimer’s disease. Glia.

[CR115] Strobel S, Grünblatt E, Riederer P, Heinsen H, Arzberger T, Al-Sarraj S, Troakes C, Ferrer I, Monoranu CM (2015). Changes in the expression of genes related to neuroinflammation over the course of sporadic Alzheimer's disease progression: CX3CL1, TREM2, and PPARγ. J Neural Transm.

[CR116] Stuart MJ, Baune BT (2014). Chemokines and chemokine receptors in mood disorders, schizophrenia, and cognitive impairment: a systematic review of biomarker studies. Neurosci Biobehav Rev.

[CR117] Sui Y, Zhang Y, Dong C, Xu B, Sun X (2019). The small molecular CCR3 antagonist YM344031 attenuates neurodegenerative pathologies and improves learning and memory performance in a mouse model of Alzheimer's disease. Brain Res.

[CR118] Thirumangalakudi L, Yin L, Rao HV, Grammas P (2007). IL-8 induces expression of matrix metalloproteinases, cell cycle and pro-apoptotic proteins, and cell death in cultured neurons. J Alzheimers Dis.

[CR119] Toepper M (2017). Dissociating Normal Aging from Alzheimer’s disease: a view from cognitive neuroscience. J Alzheimer’s Dis.

[CR120] Tripathy D, Thirumangalakudi L, Grammas P (2010). RANTES upregulation in the Alzheimer's disease brain: a possible neuroprotective role. Neurobiol Aging.

[CR121] Vacinova G, Vejražkova D, Rusina R, Holmerová I, Vaňková H, Jarolímová E, Včelák J, Bendlová B, Vaňková M (2021). Regulated upon activation, normal T cell expressed and secreted (RANTES) levels in the peripheral blood of patients with Alzheimer's disease. Neural Regen Res.

[CR122] van der Flier WM, Pijnenburg YAL, Fox NC, Scheltens P (2011). Early-onset versus late-onset Alzheimer’s disease: the case of the missing APOE ε4 allele. Lancet Neurol.

[CR123] Villeda SA, Luo J, Mosher KI, Zou B, Britschgi M, Bieri G, Stan TM, Fainberg N, Ding Z, Eggel A, Lucin KM, Czirr E, Park JS, Couillard-Després S, Aigner L, Li G, Peskind ER, Kaye JA, Quinn JF, Galasko DR, Xie XS, Rando TA, Wyss-Coray T (2011). The ageing systemic milieu negatively regulates neurogenesis and cognitive function. Nature.

[CR124] Walker DG, Lue LF, Beach TG (2001). Gene expression profiling of amyloid beta peptide-stimulated human post-mortem brain microglia. Neurobiol Aging.

[CR125] Wang Q, Xu Y, Chen JC, Qin YY, Liu M, Liu Y, Xie MJ, Yu ZY, Zhu Z, Wang W (2012). Stromal cell-derived factor 1α decreases β-amyloid deposition in Alzheimer's disease mouse model. Brain Res.

[CR126] Watson K, Fan GH (2005). Macrophage inflammatory protein 2 inhibits β-amyloid peptide (1–42)-mediated hippocampal neuronal apoptosis through activation of mitogen-activated protein kinase and phosphatidylinositol 3-kinase signaling pathways. Mol Pharmacol.

[CR127] Westin K, Buchhave P, Nielsen H, Minthon L, Janciauskiene S, Hansson O (2012). CCL2 is associated with a faster rate of cognitive decline during early stages of Alzheimer's disease. PLoS ONE.

[CR128] WHO (2020) Global health estimates. The top 10 causes of death. https://www.who.int/news-room/fact-sheets/detail/the-top-10-causes-of-death Accessed on 1 December 2021

[CR129] WHO (2021) World failing to address dementia challenge. https://www.who.int/news/item/02-09-2021-world-failing-to-address-dementia-challenge Accessed on 1 December 2021

[CR130] Winter AN, Subbarayan MS, Grimmig B, Weesner JA, Moss L, Peters M, Weeber E, Nash K, Bickford PC (2020). Two forms of CX3CL1 display differential activity and rescue cognitive deficits in CX3CL1 knockout mice. J Neuroinflammation.

[CR131] Wojta KJ, Ayer AH, Ramos EM, Nguyen PD, Karydas AM, Yokoyama JS, Kramer J, Lee SE, Boxer A, Miller BL, Coppola G (2020). Lack of association between the CCR5-*delta*32 polymorphism and neurodegenerative disorders. Alzheimer Dis Assoc Disord.

[CR132] Wu CC, Wang IF, Chiang PM, Wang LC, Shen CJ, Tsai KJ (2017). G-CSF-mobilized bone marrow mesenchymal stem cells replenish neural lineages in Alzheimer's disease mice via CXCR4/SDF-1 chemotaxis. Mol Neurobiol.

[CR133] Wu J, Bie B, Yang H, Xu JJ, Brown DL, Naguib M (2013). Suppression of central chemokine fractalkine receptor signaling alleviates amyloid-induced memory deficiency. Neurobiol Aging.

[CR134] Xia M, Hyman BT (2002). GROα/KC, a chemokine receptor CXCR2 ligand, can be a potent trigger for neuronal ERK1/2 and PI-3 kinase pathways and for tau hyperphosphorylation—a role in Alzheimer's disease?. J Neuroimmunol.

[CR135] Xia M, Qin S, McNamara M, Mackay C, Hyman BT (1997). Interleukin-8 receptor B immunoreactivity in brain and neuritic plaques of Alzheimer's disease. Am J Pathol.

[CR136] Xia MQ, Bacskai BJ, Knowles RB, Qin SX, Hyman BT (2000). Expression of the chemokine receptor CXCR3 on neurons and the elevated expression of its ligand IP-10 in reactive astrocytes: in vitro ERK1/2 activation and role in Alzheimer's disease. J Neuroimmunol.

[CR137] Xia MQ, Qin SX, Wu LJ, Mackay CR, Hyman BT (1998). Immunohistochemical study of the beta-chemokine receptors CCR3 and CCR5 and their ligands in normal and Alzheimer's disease brains. Am J Pathol.

[CR138] Xiang Y, Xin J, Le W, Yang Y (2020). Neurogranin: a potential biomarker of neurological and mental diseases. Front Aging Neurosci.

[CR139] Yamamoto M, Horiba M, Buescher JL, Huang D, Gendelman HE, Ransohoff RM, Ikezu T (2005). Overexpression of monocyte chemotactic protein-1/CCL2 in β-amyloid precursor protein transgenic mice show accelerated diffuse beta-amyloid deposition. Am J Pathol.

[CR140] Zaheer S, Thangavel R, Wu Y, Khan MM, Kempuraj D, Zaheer A (2013). Enhanced expression of glia maturation factor correlates with glial activation in the brain of triple transgenic Alzheimer's disease mice. Neurochem Res.

[CR141] Zhang K, Tian L, Liu L, Feng Y, Dong YB, Li B, Shang DS, Fang WG, Cao YP, Chen YH (2013). CXCL1 contributes to β-amyloid-induced transendothelial migration of monocytes in Alzheimer's disease. PLoS ONE.

[CR142] Zhang L, Xu J, Gao J, Wu Y, Yin M, Zhao W (2018). CD200-, CX3CL1-, and TREM2-mediated neuron-microglia interactions and their involvements in Alzheimer's disease. Rev Neurosci.

[CR143] Zhang XF, Zhao YF, Zhu SW, Huang WJ, Luo Y, Chen QY, Ge LJ, Li RS, Wang JF, Sun M, Xiao ZC, Fan GH (2015). CXCL1 triggers caspase-3 dependent tau cleavage in long-term neuronal cultures and in the hippocampus of aged mice: implications in Alzheimer's disease. J Alzheimers Dis.

[CR144] Zhao Q-F, Tan L, Wang H-F, Jiang T, Tan M-S (2016). The prevalence of neuropsychiatric symptoms in Alzheimer's disease: systematic review and meta-analysis. J Affect Disord.

[CR145] Zhao T, Su Z, Li Y, Zhang X, You Q (2020). Chitinase-3 like-protein-1 function and its role in diseases. Sig Transduct Target Ther.

[CR146] Zhou M, Greenhill S, Huang S, Silva TK, Sano Y, Wu S, Cai Y, Nagaoka Y, Sehgal M, Cai DJ, Lee YS, Fox K, Silva AJ (2016). CCR5 is a suppressor for cortical plasticity and hippocampal learning and memory. Elife.

[CR147] Zhu C, Xu B, Sun X, Zhu Q, Sui Y (2017). Targeting CCR3 to reduce amyloid-β production, tau hyperphosphorylation, and synaptic loss in a mouse model of Alzheimer's disease. Mol Neurobiol.

[CR148] Zhu M, Allard JS, Zhang Y, Perez E, Spangler EL, Becker KG, Rapp PR (2014). Age-related brain expression and regulation of the chemokine CCL4/MIP-1β in APP/PS1 double-transgenic mice. J Neuropathol Exp Neurol.

